# Applicability of L-Band and C-Band InSAR for Detecting Slow-Moving Landslides in Vegetated Tropical Andes

**DOI:** 10.3390/s26196094

**Published:** 2026-09-25

**Authors:** Emanuel Castillo-Cardona, Stefania Valencia-Herrera, Exneyder A. Montoya-Araque, Marco F. Gamboa-Ramirez, Adriana I. Osorio-Mosquera, Daniel F. Ruiz, Alejandro Marulanda-Tobon

**Affiliations:** 1 Proyecto Sistema de Alerta y Monitoreo de Antioquia (SAMA), Universidad EAFIT, Carrera 49 N° 7 Sur-50, Medellín 050022, Colombia; svalenciah@eafit.edu.co (S.V.-H.); eamontoyaa@eafit.edu.co (E.A.M.-A.); mgamboa@eafit.edu.co (M.F.G.-R.); 2 School of Applied Sciences and Engineering, Universidad EAFIT, Carrera 49 N° 7 Sur-50, Medellín 050022, Colombia; aiosoriom@eafit.edu.co (A.I.O.-M.); dfruizr@eafit.edu.co (D.F.R.); amarula2@eafit.edu.co (A.M.-T.)

**Keywords:** landslide, time-series InSAR, remote sensing, tropical region

## Abstract

Interferometric Synthetic Aperture Radar (InSAR) enables the measurement of ground deformation with high spatial coverage and frequent temporal sampling, making it a valuable tool for landslide monitoring in remote mountainous regions. However, in tropical mountainous environments, dense vegetation, steep topography, and rapid surface changes often reduce interferometric coherence, limiting the reliability of C-band observations. This study evaluates the applicability of C-band and L-band spaceborne InSAR data for detecting slow-moving landslides in the northern tropical Andes. The analysis focuses on two case studies in the northern Colombian Andes, where slow-moving landslides have affected two municipalities. Time-series deformation and velocity analysis were derived using a Small Baseline Subset (SBAS) approach applied to Sentinel-1 (C-band) and SAOCOM-1 (L-band) SAR datasets. The detected deformation patterns were compared with independent field evidence reported in previous geotechnical investigations of the study area. Results show that both sensors successfully identified the spatial extent of the slow-moving landslide. However, L-band data exhibited higher coherence and reduced noise levels in densely vegetated zones. These differences highlight the improved performance of L-band observations under tropical vegetation conditions, while C-band data remain effective for detecting moderate surface displacements where coherence is preserved. The findings provide guidance for selecting appropriate SAR wavelengths for landslide detection in tropical mountainous regions and support the integration of multi-frequency InSAR for operational hazard assessment.

## 1. Introduction

Landslides represent one of the most widespread geomorphological hazards in tropical mountainous regions, where intense rainfall and steep topography frequently trigger slope instabilities [1,2]. Even though slow-moving landslides are common in these environments, accurately identifying their spatial distribution remains difficult, especially in remote or topographically complex areas that limit systematic field surveys [3,4]. Such landslides are especially concerning when they occur near urbanized areas, as they pose direct risks to human life while simultaneously disrupting everyday functioning and obstructing the flow of goods and services, leading to significant indirect economic impacts [5].

Among the various types of slope failures, slow-moving landslides are especially critical due to their progressive evolution and tendency to remain unnoticed until substantial damage occurs [6]. Their detection is particularly challenging because pre-failure displacements generally occur at very low rates, on the order of centimeters per year [7], and the practical limitations of investigations in mountainous terrains hinder timely recognition and early warning efforts [8]. Traditional ground-based techniques for detecting landslide surface deformation, such as total stations, leveling surveys, and GPS observations, offer high precision but require direct field deployment, resulting in considerable logistical effort and expense [9,10]. In addition, these approaches are generally restricted to localized measurements at known unstable sites and are not suitable for systematic, wide-area detection of unidentified or emerging landslides [11].

Over the past two decades, satellite-based tools have significantly transformed landslide identification, enabling more efficient spatial and temporal detection [12]. Among these, Synthetic Aperture Radar (SAR) is widely used for detecting gravitational slope deformation, including both rapid failures [13,14] and slow-moving landslides [15,16], with InSAR in particular enabling millimetric ground deformation measurements across extensive regions through all-weather, day-and-night, cloud-penetrating acquisitions [17,18]. However, the applicability of InSAR in tropical Andean environments is often challenged by dense vegetation cover, high humidity, and rapid surface changes that significantly reduce interferometric coherence, particularly in X- and C-band data [19]. Time-series approaches such as the Small Baseline Subset (SBAS) method [18,20,21] partially mitigate these decorrelation issues and have been successfully implemented across tropical and subtropical regions with persistent vegetation cover, including Southeast Asia, the Indian subcontinent, Africa, and South America [14,16,22]. Despite these advances, significant challenges remain for reliably detecting landslides in densely vegetated areas due to temporal decorrelation, geometric distortions, and signal attenuation effects [4].

Although L-band SAR systems are theoretically less affected by temporal decorrelation in vegetated areas than C-band systems [4,23], their comparative applicability for detecting slow-moving landslides in tropical Andean settings remains insufficiently explored. Previous studies have reported enhanced coherence and deformation retrieval capabilities of L-band data over forested terrains [23,24], as well as differences in geometric distortions and displacement sensitivity between wavelengths [4]. However, many of these assessments have been conducted in managed forests, temperate regions, or for potential landslide mapping rather than for rainfall-triggered slow-moving landslides in highly dynamic tropical Andean settings. In particular, limited research has examined the multi-temporal applicability of L-band and C-band SBAS-InSAR approaches under conditions of persistent cloud cover, dense natural vegetation, and rapid surface moisture changes typical of the northern tropical Andes. Therefore, a systematic evaluation of the applicability of both wavelengths for detecting and delineating slow-moving deformation in this environment is still needed.

This study investigates rainfall-triggered slow-moving landslides in the northern Colombian mountainous terrain, focusing on two areas with complex geomorphological settings [25,26], to evaluate the performance of multi-frequency InSAR observations under vegetated conditions. The tropical Andes are characterized by intense seasonal precipitation [27,28] and highly weathered soils [29], conditions that promote recurrent slope instability. In this region, extreme rainfall events constitute the dominant triggering mechanism for both rapid and slow-moving landslides [27]. The combination of persistent vegetation cover, strong temporal fluctuations in soil moisture, and complex topography creates conditions that frequently degrade interferometric coherence in tropical mountainous environments. The selected cases, therefore, provide a well-constrained temporal framework for deformation analysis, offering an ideal setting to evaluate InSAR performance under challenging conditions.

Building upon this context, this study aims to evaluate the applicability of multi-temporal InSAR derived from Sentinel-1 (C-band) and SAOCOM-1 (L-band) data for detecting and delineating slow-moving landslides in densely vegetated tropical mountains. The research focuses on analyzing differences in coherence results, deformation, geometric distortions, and the impact of vegetation-related temporal decorrelation on the interferometric signal. The InSAR-derived deformation patterns are further examined against independent geomorphological and field-based evidence to assess their consistency and reliability. By doing so, this work seeks to clarify the conditions under which each wavelength provides robust information, contributing to informed SAR data selection strategies for landslide monitoring in the tropical Andes.

## 2. Study Area

This study focuses on the northern Colombian Andes, specifically on two mountainous sectors located in the municipalities of Venecia and Valparaíso in the department of Antioquia, Colombia (Figure 1). The investigated sectors are located near the urban centers of both municipalities (Figure 1b,c), while their regional location within northwestern South America is shown in Figure 1c. The municipality of Venecia covers 141 km^2^ and is characterized by steep mountainous terrain, with elevations ranging from 537 to 2587 m above sea level, a vertical difference of about 2000 m. Valparaíso extends over 130 km^2^ and presents similar mountainous physiography, with an average elevation of about 1375 m above sea level. In both municipalities, the topography exhibits a pronounced relief typical of the northern Colombian Andes, with deeply incised valleys and elevated ridges shaping the landscape.

Both municipalities concentrate population and assets in settings directly exposed to slope instability. Venecia has approximately 12,300 inhabitants, of which 57% reside in the rural area and 43% in the urban center, which comprises 38 urban blocks; the rural population is distributed among several hundred dispersed dwellings built on the surrounding hillslopes [30]. Valparaíso has approximately 6700 inhabitants with the opposite distribution, 56% urban and 44% rural, organized in 40 urban blocks and several tens of dispersed rural dwellings [30]. Connectivity in both municipalities depends on a single road corridor. Venecia, 60 km from Medellín (the second largest city of Colombia), is connected to neighboring municipalities through one main road, while access to Valparaíso, 98 km from Medellín, relies on the intermunicipal route through La Pintada. In both cases the interruption of that corridor isolates the urban center together with the rural settlements that depend on it. The rural network consists of paved narrow tracks following the hillslopes, which provide the only access to the dispersed dwellings and to the productive areas. Economic activity is predominantly agricultural and rests entirely on this network. In Venecia it is based on coffee, plantain, cassava and citrus cultivation, cattle and pig farming, and coal mining in the Amagá-Venecia sector. In Valparaíso, cattle ranching is the dominant activity, complemented by sugarcane, beans, coffee, cacao and maize, and dairy products. All these activities are developed on hillslopes and depend on road transport to reach processing and market centers.

Essential services in both towns are concentrated within or immediately adjacent to the urban perimeter and are therefore exposed to the same slopes of the mapped landslides. Water supply in both municipalities depends on gravity-fed systems that capture surface water from headwater streams on the surrounding hillslopes and convey it to storage tanks located at the upper part of each urban center, a configuration that places both the conduction lines and the storage facilities on terrain susceptible to displacement. Electricity is delivered through a voltage distribution line that follows the main road corridor into each town and is then distributed to the rural area by overhead lines supported on poles installed along the tracks. Both the conduction of water and the distribution of power therefore share the corridors that the landslides intersect so that a single displacement episode can simultaneously affect access, water supply, and electricity for the urban center and for the dispersed rural settlements that depend on it.

Both areas are located within a humid tropical climatic regime characterized by high annual precipitation and a bimodal rainfall pattern typical of the central Colombian Andes. This precipitation regime is largely controlled by the meridional migration of the Intertropical Convergence Zone (ITCZ) and the influence of the Chocó low-level jet [31,32]. Seasonal rainfall variability plays a critical role in triggering landslides throughout mountainous regions of Colombia, particularly within the central Andean zone [33]. As a result, hillslopes in these municipalities frequently experience rainfall-induced instability.

### 2.1. Geological Framework

The northern Colombian Andes are divided into three independent mountain systems known as the Western, Central, and Eastern Cordilleras. This mountain belt results from the complex interaction between the South American Plate and the subducting Nazca and Caribbean plates. The resulting tectonic framework has produced a structurally controlled mountainous region composed of active fault systems, crustal shortening, uplift, and significant seismic activity [34,35]. The study areas are located within the northern segment of the Central (Venecia) and Western (Valparaiso) Cordillera of Colombia, which forms part of the northwestern branch of the Andean orogenic system. The Central Cordillera is predominantly composed of Paleozoic–Mesozoic metamorphic and igneous basement rocks intruded by granitoids and locally overlain by Cenozoic volcanic and volcaniclastic sequences [36]. The Western Cordillera, in turn, comprises a sequence of Cretaceous and Paleogene volcanic rocks and marine sediments, intruded and overlain by Neogene igneous rocks and volcanic sequences [37,38]. In the northern segment of this Cordilleran system, these basement rocks are unconformably overlain by a clastic sedimentary succession assigned to the Amagá Formation, an upper Oligocene-middle Miocene siliciclastic and coal-bearing sequence of conglomerates, sandstones, and mudstones accumulated in fluvial systems within intramontane pull-apart basins along the Cauca Valley [39,40,41].

Both slow-moving landslides developed on the same geological unit, the Amagá Formation, whereas the steeper slopes surrounding them are underlain by andesitic porphyries of the Combia Formation (Figure 2). The Valparaíso study area is located in a sector dominated by deposition driven by the increase in erosion associated with the continuous Cenozoic uplift of the Central Cordillera, which generated uplift to the Oligocene-Miocene sedimentary sequence of the Amagá Formation [39]. The material involved in the movement corresponds to continental claystones, siltstones, and sandstones of the upper member of the unit, accumulated in meandering and braided piedmont fluvial systems. These strata dip towards the northwest and define a prominent structural cuesta north of the main urban center, along which the landslide has developed, and are intruded by andesitic porphyritic bodies of the Miocene Combia Formation [42]. At the Venecia study area, in contrast, the Amagá Formation comprises mainly interbedded claystones and mudstones of dark reddish-brown to brown colour, arranged in thin to medium tabular beds with plane-parallel to wavy geometry, with sporadic intercalations of well-sorted fine- to medium-grained sandstones of greenish-grey to dark grey colour, in medium tabular beds not exceeding 3 m in thickness.

### 2.2. Geomorphological Characterization and Land Surface Process

The terrain morphology of the two areas is similar; the dominant presence of sedimentary and volcano-sedimentary lithological units generated low-gradient slopes with smoothed morphologies and a structural control associated with bedding (Figure 3). The structural geology of the study areas is governed by the dip of the Amagá Formation strata; however, the intense weathering typical of tropical settings makes it difficult to obtain clear evidence supporting the presence of a structural slope beneath the residual soils. Previous studies of the Amagá Formation report tilting of the strata and propose the hypothesis of a possible paleorelief in certain sectors that could generate instability [43], although the available data are insufficient to confirm this. Furthermore, no clear expressions of deformation associated with regional faulting were identified in any of the geological units cropping out in the study area, considering that such evidence was possibly buried by the Amagá Formation.

Geomorphologically, gently inclined slopes of this type, developed on clastic sedimentary rocks and oriented in the direction of dip, are classified as structural cuestas in the geomorphological maps produced by local institutions [43]. In this context, the slow-moving landslides may result from the instability generated by the preferential northward orientation of the Amagá Formation strata, since the displacements in both study areas share the same northward direction, along which the slope angle simultaneously decreases.

At Venecia, the landslide may reach the urban centre and affect it considerably. The movement is mapped over an extent of 700 m in length and 400 m in width at its widest sector. Its depth is estimated at 30 m, and 63 isolated cracks have been reported [44], although the available data are insufficient to delineate a main scarp. At Valparaíso, the landslide extends parallel to the urban center, affecting the road and community infrastructure. Two landslides were identified in this study area; the larger one extends for 2800 m and is 700 m wide, while the second extends for 1500 m and is 400 m wide. Both have an estimated depth of 25 m and more than 300 recorded cracks [45]. The failure mechanism is similar in both study areas, as they share the same lithology, geomorphology, slope angle and smoothed relief morphology. At Valparaíso, the failure mechanism may be associated with a complex translational rupture controlled by bedding along a dip slope. At Venecia, the mechanism also shows translational kinematic characteristics owing to the influence of the Amagá Formation, but differs in that additional processes remain active in the area: undercutting by the fluvial dynamics of the adjacent drainages may be eroding the toe of the movement, unconfining its base and thereby activating displacement. In addition, this event occurred during a debris flow in the upper part of the movement, which may have loaded the slope and reactivated the displacement.

### 2.3. Occurrence of Slow-Moving Landslides

The combined effects of the climatic, geological, and geomorphological conditions described above have led to the recurrent occurrence of landslides and related hazards in both municipalities. In the municipality of Valparaíso, a significant mass movement was reported on 10 August 2022 (Figure 4). The event affected the functionality of the intermunicipal road connecting Valparaíso and La Pintada, isolating the town. The slow-moving landslide also compromised the habitability of several nearby houses. Due to the magnitude of the impacts, the local government declared a public calamity emergency.

In Venecia, an intense nighttime rainfall event reaching nearly 80 mm $h^{-1}$ triggered the reactivation of a slow-moving slope near the urban center on 25 June 2024. The resulting landslide affected 20 houses, blocked the main road connecting the town with neighbouring municipalities, fractured the drinking water supply tank of the town, and caused two fatalities, highlighting the persistent vulnerability of settlements located near unstable slopes (Figure 5).

## 3. Data and Methods

### 3.1. Datasets

In this study, we used two SAR datasets acquired over the same observation period to exploit the complementary sensitivity of C- and L-band radar to ground deformation and decorrelation. We used descending Sentinel-1 Interferometric Wide Swath (IW) data in the C-band, and descending SAOCOM-1 Stripmap (SM) data in the L-band. The Sentinel-1 products were acquired in dual-polarization mode, whereas the SAOCOM-1 products were acquired in quad-polarization mode. However, only the co-polarized VV channel was selected from both datasets for interferogram generation and subsequent SBAS processing, ensuring consistency in the interferometric comparison between the two sensors. Sentinel-1 is a two-satellite constellation; each satellite has a nominal 12-day repeat cycle, and the combined constellation enables an effective revisit of 6 days. SAOCOM-1 operates with a nominal 16-day repeat cycle per satellite; however, the effective revisit time used in this study is 8 days (see Table 1), resulting from the combined operation of both satellites of the constellation (SAOCOM-1A and 1B). As an L-band SAR system, SAOCOM-1 is designed for all-weather observations, including conditions with strong atmospheric variability.

A total of 58 Sentinel-1 scenes and 32 SAOCOM-1 scenes were processed, covering the period from January 2024 to August 2025. For Sentinel-1, interferometric network generation was constrained using a temporal baseline threshold of 60 days and a spatial baseline threshold of 180 m, resulting in 275 interferograms for time-series processing (Figure 6a). For SAOCOM-1, a temporal baseline threshold of 180 days and a spatial baseline threshold of 2000 m were applied, yielding 243 interferometric pairs (Figure 6b). To remove topographic phase contributions and support accurate geocoding, the Shuttle Radar Topography Mission (SRTM) DEM [46] was used as the reference elevation model.

The time-series deformation products derived for both SAOCOM-1 and Sentinel-1 in this study were obtained with the Small Baseline Subset (SBAS) approach, originally formulated by Berardino [18] and subsequently consolidated as a standard tool for resolving non-linear surface motion from stacks of short-baseline interferograms [47,48]. By constraining interferometric pairs to short temporal and spatial baselines, SBAS limits decorrelation while preserving network connectivity, which is precisely what allows it to track the irregular, episodic kinematics typical of landslide-affected slopes rather than a single constant rate [49,50]. The same inversion strategy has recently been applied to comparably steep, geomorphologically active settings, including reservoir-bank landslides in the Three Gorges area [51], long-term basin-scale monitoring in the Xiaojiang River Basin [52], and C/L-band comparisons in steep mountainous catchments of southwest China [4].

The processing chain implemented in this work is organized into three sequential stages, preprocessing, SBAS processing, and postprocessing, summarized in Figure 7. Each stage consumes the outputs of the preceding one, which keeps the chain traceable from the raw SAR acquisitions to the interpreted deformation products.

### 3.2. Preprocessing

The preprocessing stage transforms the raw Single Look Complex (SLC) acquisitions into a coregistered, coherent stack of interferograms, and was carried out independently for the Sentinel-1 and SAOCOM-1 datasets.

Candidate image pairs were first screened using sensor-specific temporal and perpendicular-baseline thresholds selected to limit decorrelation while preserving a well-connected interferometric network. For Sentinel-1, thresholds of 60 days and 180 m were adopted, in agreement with the dense temporal sampling of the sensor and with threshold ranges commonly reported in landslide-oriented studies [4,51]. For SAOCOM, less restrictive thresholds of 180 days and 2000 m were applied to compensate for the lower acquisition frequency and to avoid excessive fragmentation of the interferometric network. After this initial screening, only interferometric pairs with a mean coherence equal to or greater than 0.4 were retained for the subsequent processing steps.

Differential interferograms were then generated from the retained pairs, removing the topographic phase contribution with the 1 arc-second SRTM DEM. Spatial multilooking reduced speckle noise, yielding effective pixel sizes of 32 m for Sentinel-1 and 48 m for SAOCOM-1; because coherence estimation and speckle reduction depend directly on the number of looks used, this resolution difference is expected to influence the apparent coherence and the visual continuity of the deformation maps independently of the operating wavelength.

SAOCOM-1 data were processed with the InSAR Scientific Computing Environment (ISCE) [53], with the resulting unwrapped interferograms subsequently inverted using MintPy for time-series analysis, while Sentinel-1 SLC bursts were processed with pyGMTSAR [54]. Sentinel-1 SLC bursts are readily available through the Alaska Satellite Facility (ASF) Search platform, which allowed the interferometric stack to be generated efficiently using the pyGMTSAR notebook-based workflow, whereas SAOCOM-1 SLC data are not distributed through ASF and require interferogram generation through the ISCE processing chain. While both pipelines implement the same underlying SBAS formulation, differences in default processing parameters, filtering strategies, and multilooking implementations between pyGMTSAR and ISCE/MintPy cannot be fully ruled out as contributing factors to the differences observed between the two datasets.

Phase unwrapping followed the 3D Minimum Cost Flow (MCF) algorithm [55,56,57], which resolves the 2π ambiguity of the wrapped phase by minimizing a global cost function over the interferometric network; this gives a spatially consistent solution even where decorrelation is locally severe. The unwrapped interferograms were finally assembled into the stack that feeds the SBAS inversion in Step 2.

### 3.3. SBAS Processing

The SBAS stage converts the interferometric phase observations into a temporally ordered displacement history for each coherent pixel, following the formulation originally introduced by Berardino [18] and subsequently consolidated in later implementations [4,47,48,52]. Briefly, the unwrapped phase of each interferogram is expressed as the difference between the deformation phase at the two acquisition dates, plus residual contributions from topography, atmosphere, and decorrelation noise. Because these non-deformation components are not necessarily random—atmospheric delays, orbital ramps, and residual topographic errors may exhibit spatial or temporal structure—they were estimated and mitigated during the inversion and post-processing stages rather than being treated only as uncorrelated noise.

Rather than solving directly for absolute phase, the interferometric network was reformulated in terms of the mean deformation velocity between consecutive acquisition dates, following the standard approach used to handle irregularly connected networks [18,47,48]. For the complete interferometric stack, this relation is written as

(1) Bv=δφ,

where v contains the unknown interval velocities and B is an M×N matrix whose non-zero elements are the time intervals spanned by each interferogram.

Because the interferometric network may contain redundant observations or disconnected subsets, the resulting system is not always directly invertible; it was therefore solved in a least-squares sense using Singular Value Decomposition (SVD), which provides a stable minimum-norm solution even when the design matrix is ill-conditioned or rank-deficient. The cumulative displacement time series was then obtained by integrating the estimated interval velocities through time, and the radar-coordinate outputs were geocoded into a geographic reference system to produce the deformation maps used for interpretation.

Before deriving the final velocity and displacement products, two additional corrections were applied. First, residual topographic phase, identified through its relationship with the perpendicular baseline and mainly attributed to SRTM DEM errors, was estimated and removed together with residual orbital ramps. Second, tropospheric phase delays were mitigated using the Generic Atmospheric Correction Online Service for InSAR (GACOS), based on the Iterative Tropospheric Decomposition (ITD) model [58].

After these corrections, the mean line-of-sight velocity for each pixel was calculated as the slope of a linear fit to the corrected displacement time series. Acquisitions identified as unreliable during quality control were excluded from this estimation. Cumulative displacement was obtained by integrating the corrected velocity field over the observation period. Both products were exported in geocoded geographic coordinates and used as the main inputs for the spatial and temporal analysis described below.

### 3.4. Postprocessing

The postprocessing stage included the development of (i) displacement maps and independent field evidence; (ii) time series of accumulated displacement and precipitation; (iii) coherence and land cover maps; and (iv) geometric distortions.

#### 3.4.1. Displacement Maps and Independent Field Evidence

The velocity and cumulative displacement fields were then clipped to the boundaries of each study area. The raster cells were subsequently converted into point features located at the center of each pixel. Each point was symbolized according to its cumulative LOS displacement using a common colour scale for both sensors and both study areas. The resulting point layers were overlaid on high-resolution optical imagery to provide a geographic reference.

Field observations were used as independent supporting evidence for interpreting the displacement results. The available field evidence consisted mainly of the extension of slow-moving landslides and surface cracks in the affected areas. Since no systematic ground-based displacement measurements was available, these observations were not used as a quantitative validation dataset. Instead, they served to assess whether the spatially coherent deformation patterns detected by InSAR were consistent with visible signs of ground instability. These external datasets provided a detailed reference of slope instability, allowing the satellite-derived deformation patterns to be interpreted in the context of known ground conditions and reported slow-moving landslides.

#### 3.4.2. Time Series of Accumulated Displacement and Precipitation

Time series of accumulated displacement were extracted to examine the temporal behavior of the movements in relation to rainfall. For each acquisition date, the LOS displacement values of all coherent pixels falling within the mapped landslide extent were averaged. This spatial averaging was applied independently to the Sentinel-1 and SAOCOM-1 stacks, each on its own acquisition dates and native pixel spacing, and the resulting series were referenced to the first acquisition of each stack.

On the other hand, rainfall data were obtained from the nearest gauge station with continuous records covering the observation period. Daily accumulated precipitation was used, and the series was neither interpolated nor gap-filled. The rainfall record was plotted on a secondary vertical axis together with the displacement series, so that the timing of precipitation events could be compared directly with the evolution of the accumulated displacement at each site.

#### 3.4.3. Coherence and Land Cover Maps

Interferometric coherence was used as a complementary quality metric, defined as the normalized cross-correlation between two co-registered SAR images, with magnitude |γ| ranging from 0 (decorrelated) to 1 (stable scattering). Assuming $S_{1}$ and $S_{2}$ are two co-registered, equally polarized SAR images,

(2) $$\gamma =\frac{\langle S_{1}S_{2}^{*}\rangle }{\sqrt{\langle |S_{1}{|}^{2}\rangle \langle |S_{2}{|}^{2}\rangle }},$$

where 〈·〉 denotes local spatial averaging or speckle filtering, ∗ is the complex conjugate, |γ| represents the magnitude of the correlation, and arg(γ) corresponds to the interferometric phase [59]. In practice, coherence was estimated pixel by pixel over a moving window, equivalent to the discrete implementation commonly used in InSAR studies [4]. High coherence is generally associated with stable scatterers such as exposed rock, bare soil, or built-up surfaces, whereas vegetation, water, and rapidly changing surfaces tend to reduce phase correlation [60]. A minimum coherence threshold of 0.4 was adopted to mask unreliable pixels before interpretation. This value lies within the range commonly used in landslide studies, where thresholds between 0.3 and 0.6 are often selected to balance spatial coverage and measurement reliability [51,61].

To characterize the vegetation conditions of the two study areas, a land cover map was produced for each site from Sentinel-2 Level-2A surface reflectance imagery through unsupervised classification. Cloud-free composites were built by masking each scene, using acquisitions between June and September 2024 for both study areas. Six spectral bands (blue, green, red, near infrared, and the two shortwave infrared bands) were combined with the Normalized Difference Vegetation Index, the Normalized Difference Built-up Index, the Normalized Difference Water Index, the Bare Soil Index, and overall brightness. These variables were classified with a k-means algorithm at 10 m resolution. The classification was partitioned into four spectral clusters, corresponding to the dominant covers of both study areas: urban, grassland, sparse vegetation, and dense forest. The fraction of each class was quantified separately for the total study area and for the mapped landslide extent.

Coherence values were then grouped by land cover category and summarized through their median and interquartile range, both over the total study area and within the landslide extent. Two spatial scopes were analyzed separately: the total study area, which provides the sample size required to characterize the coherence-land cover relationship, and the landslide extent, which indicates the land cover conditions in the deformation results.

#### 3.4.4. Geometric Distortions

In addition, radar geometric distortion was explicitly considered because the side-looking acquisition geometry can limit the reliability of InSAR measurements in steep terrain. Areas affected by foreshortening, layover, and radar shadow were identified on a per-pixel basis by combining the SRTM DEM with the acquisition geometry of each sensor (incidence angle, look direction, and orbit heading), following the rationale used in previous C- and L-band assessments over mountainous areas [4].

Two complementary criteria were used to build the distortion mask. First, a layover–shadow mask was derived from the DEM and the sensor viewing geometry, identifying pixels affected by shadow, by the joint occurrence of layover and shadow, and by layover alone. Second, for pixels not already flagged by this mask, the projected local incidence angle, the DEM-derived local incidence angle projected onto the sensor line of sight, was compared against the incidence angle that would result from a smooth ellipsoidal reference surface at the same location. Where the projected local incidence angle was smaller than the ellipsoid-based incidence angle, the pixel was classified as affected by foreshortening, since this condition indicates that the local slope compresses the illuminated ground range relative to a flat reference.

These two criteria were combined into a single four-class distortion mask, applied per pixel with the following priority: pixels flagged as shadow, or as a joint occurrence of layover and shadow, were assigned to the shadow class; remaining pixels flagged as layover were assigned to the layover class; remaining pixels whose projected local incidence angle was smaller than the ellipsoid-based incidence angle were assigned to the foreshortening class; and all other pixels were classified as suitable for observation. Unlike shadow and layover, which correspond to an actual loss or geometric inversion of the radar signal, foreshortening reflects a compression of the illuminated ground range in which the signal is still recorded and can preserve interferometric coherence, although the resulting phase represents a mixed contribution from multiple terrain points.

## 4. Results

### 4.1. Slow-Moving Landslide Displacements

Figure 8 and Figure 9 present the ground deformation patterns derived from InSAR analysis for the Valparaíso and Venecia sectors, respectively. The displacement values are expressed along the satellite line-of-sight (LOS), where negative values (red tones) indicate motion away from the sensor, positive values (blue tones) represent motion toward the sensor, and values near zero (green tones) correspond to relatively stable areas.

In Figure 8 (Valparaíso), both Sentinel-1 and SAOCOM-1 datasets reveal a well-defined deformation corridor aligned with the slope, consistent with the expected geometry of a slow-moving landslide. The Sentinel-1 results show a broader dispersion of deformation signals, with noticeable variability and localized noise, particularly in vegetated areas. In contrast, the SAOCOM-1 results exhibit a more coherent and continuous deformation pattern, allowing for a clearer delineation of the affected zone. The deformation is predominantly characterized by negative LOS values, suggesting downslope movement relative to the sensor geometry. Field observations, including mapped surface cracks, spatially coincide with the central portion of the detected deformation zone. In the northern sector of the landslide, where an extension of the movement has been reported but field evidence is limited to a smaller number of surface cracks, both Sentinel-1 and SAOCOM-1 datasets indicate deformation rates of 40 mm/year, suggesting that significant ground displacement may be occurring beyond the most visibly affected areas.

A similar pattern is observed in Figure 9 (Venecia), where the deformation field corresponds to a previously reported slow-moving landslide located near the urban boundary. The highest displacement values are concentrated within the mapped landslide body, while surrounding areas remain relatively stable. The field-based dataset, consisting of reported surface cracks and mapped landslide boundaries, shows a strong spatial agreement with the zones of highest deformation, particularly in the central and downslope sectors. In contrast, in the northern portion of the Venecia landslide—where numerous surface cracks have been reported—neither sensor clearly captures significant deformation, with only Sentinel-1 indicating localized, displacement values on the order of 40 mm/year.

Figure 10 illustrates the relationship between accumulated line-of-sight (LOS) displacement and precipitation for the Valparaíso sector. For each acquisition date, the displacement value corresponds to the spatial average of all coherent pixels within the slow-moving landslide (Figure 8). Blue circles represent Sentinel-1 SAR acquisitions and their corresponding LOS displacement (mm), and orange squares represent SAOCOM acquisitions and their LOS displacement (mm). Both Sentinel-1 and SAOCOM-1 exhibit a general trend of increasing negative displacement throughout the observation period, indicating progressive downslope movement. However, the Sentinel-1 signal appears smoother and more consistent over time, while the SAOCOM-1 measurements show higher variability, with abrupt fluctuations between consecutive acquisitions. Periods of intensified rainfall are represented by the light blue bars, with maximum peaks of 60 mm of precipitation during some days.

Figure 11 presents the equivalent analysis for the Venecia sector, also constrained to the zones identified as the slow-moving landside in Figure 9. In this case, the deformation signal is more clearly defined, with Sentinel-1 showing a steady and continuous increase in negative displacement over time. The SAOCOM-1 data follow a similar overall trend but display greater short-term variability, particularly during periods of frequent rainfall. The precipitation record indicates a marked increase in rainfall events during the second half of the observation period, with several peaks exceeding 40–60 mm.

### 4.2. Coherence Analysis

Figure 12 shows the average coherence maps for both sensors at the two study areas. At Venecia, the SAOCOM-1 map (Figure 12a) displays generally high values across the map, with a broad zone of elevated coherence extending over the urban center and the slopes immediately to the south, and lower values confined to the northern and eastern margins. The Sentinel-1 map of the same area (Figure 12b) shows a markedly more fragmented pattern, in which high coherence is restricted to compact patches coinciding with the urban center and with isolated cleared areas, while the surrounding terrain appears predominantly with low-medium coherence values. At Valparaíso, the SAOCOM-1 map (Figure 12c) again presents a spatially continuous distribution, with the highest values along the western sector and a gradual decrease towards the east. The corresponding Sentinel-1 map (Figure 12d) exhibits a granular texture; the urban center and some rural areas are the only features clearly obtaining high coherence values. In both study areas, the mapped landslide extents and the reported surface cracks fall within sectors of high coherence in the SAOCOM-1 maps, whereas in the Sentinel-1 maps they fall within sectors of intermediate coherence values. The statistics summarised in Table 2 quantify these patterns. SAOCOM-1 reaches a median coherence of 0.714 at Valparaíso and 0.751 at Venecia, against 0.415 and 0.478 for Sentinel-1. The fraction of the study area exceeding the 0.4 threshold follows the same ordering but with a wider separation: SAOCOM-1 retains 99.43% of its valid pixels at Valparaíso and 98.72% at Venecia, whereas Sentinel-1 retains 60.63% and 81.47%.

The land cover maps of both study areas are presented in Figure 13 and their composition is summarised in Table 3. Sparse vegetation is the dominant category at both sites, covering 58.1% of the total study area at Venecia and 77.3% at Valparaíso. The remaining categories are distributed differently between the two areas: at Venecia, grassland accounts for 16.0%, dense forest for 14.4% and urban for 11.5%, whereas at Valparaíso grassland and dense forest cover 9.9% and 9.5% respectively and the urban category is reduced to 3.3%.

Within the mapped extents of the landslides, the composition differs from that of the total study area. At Venecia, sparse vegetation decreases to 54.0% and dense forest rises to 30.1%, more than double its share of the total area, while urban and grassland fall to 6.6% and 9.2%. At Valparaíso, sparse vegetation retains the same proportion as in the total study area, 77.3%, and dense forest increases from 9.5% to 12.6%, with grassland and urban accounting for 8.3% and 1.7%. The landslide extents cover 19.8 ha at Venecia and 199.0 ha at Valparaíso.

The coherence distributions stratified by land cover class are shown in Figure 14. Over the total study area, SAOCOM-1 exhibits median coherence values above 0.7 in all four land cover classes at both sites, with narrow and unimodal distributions and limited variation between classes. Sentinel-1 shows different results: at Venecia (Figure 14a and Table 4) the median is highest in the urban class, at 0.76, and decreases to values close to 0.45 in sparse vegetation, 0.54 in grassland, and 0.53 in dense forest, with distributions that are markedly wider and, in the vegetated classes, concentrated towards the lower end of the range. At Valparaíso (Figure 14b) the same pattern is observed, with the urban class reaching a median of 0.63 and the vegetated classes remaining below 0.5.

Within the landslide bodies (Figure 14c,d) the distributions are narrower for both sensors, reflecting the smaller number of pixels involved. SAOCOM-1 retains medians between 0.6 and 0.77 across all classes at both sites, whereas Sentinel-1 remains below 0.55 in every class, with the lowest values in sparse vegetation.

### 4.3. Geometric Distortion Analysis

Figure 15 shows the spatial distribution of the geometric distortion classes. In Venecia (Figure 15a,b), most of the mapped landslide body and the reported surface cracks are located within areas classified as suitable for observation for both SAOCOM-1 and Sentinel-1. Foreshortening is restricted mainly to a narrow sector along the northeastern margin of the landslide, while layover and shadow are spatially limited.

In Valparaíso (Figure 15c,d), the geometric distortion pattern is more heterogeneous. Foreshortening affects a larger portion of the elongated landslide corridor, particularly in the upper and central sectors where numerous surface cracks have been reported. The Sentinel-1 classification shows a greater extent of foreshortening within these sectors, whereas SAOCOM-1 preserves suitable-observation conditions over a larger portion of the mapped landslide. Layover and shadow remain limited for both sensors.

The quantitative distribution of the geometric distortion classes is presented in Figure 16. In Venecia, both sensors show very similar distributions. Suitable-observation terrain accounts for 75% of the total study area and increases to 93–94% within the mapped landslide body. Foreshortening represents most of the remaining area, whereas layover and shadow are negligible for both sensors.

A stronger contrast between the two sensors is observed in Valparaíso. SAOCOM-1 presents a suitable-observation fraction of 64%, compared with 51% for Sentinel-1. Conversely, foreshortening affects 35% of the SAOCOM-1 observations and 48% of the Sentinel-1 observations. These proportions remain similar when considering either the total study area or only the mapped landslide body.

Foreshortening was the dominant geometric distortion in both study areas, whereas layover and shadow represented only a minor fraction of the analyzed terrain. Because large portions of the mapped landslide bodies, including sectors with reported surface cracks, were affected by foreshortening, excluding these pixels would have substantially reduced the spatial coverage available for interpretation. For this reason, pixels affected by foreshortening were retained in the deformation analysis, but were considered to have reduced geometric reliability and were interpreted with additional caution. In contrast, pixels classified as shadow or layover were excluded because these conditions correspond to an absence of useful radar illumination or to severe geometric ambiguity.

## 5. Discussion

InSAR enables the detection of ground surface deformation with millimetric precision and has proven particularly effective for identifying slow-moving and continuously deforming landslides. However, its performance is strongly influenced by environmental and geomorphological factors, including atmospheric conditions, topographic complexity, vegetation cover, and the rate of ground movement, all of which can affect phase stability and the reliability of deformation estimates [23,24]. These constraints have motivated a growing body of comparative work contrasting SAR bands and platforms in steep, vegetated terrain [4,52], which offers a useful external benchmark for interpreting the SAOCOM-1/Sentinel-1 contrast discussed below.

Before presenting this comparison, it is important to note that the Sentinel-1 and SAOCOM-1 datasets used in this study differ not only in operating wavelength but also in several other characteristics that can independently influence the derived products: the number of available scenes, the temporal and spatial baseline thresholds used to construct the interferometric network, the effective pixel size after multilooking, and the processing software employed. Because a coarser pixel size generally increases the number of looks used in coherence estimation and produces smoother, more spatially averaged deformation fields, part of the improved coherence and spatial continuity observed for SAOCOM-1 may be attributable to its coarser resolution rather than exclusively to the canopy-penetration advantage of L-band. Consequently, while the convergence with the independent findings of [4,52] supports a genuine wavelength-dependent coherence advantage in vegetated terrain, the present comparison should be regarded as indicative rather than fully controlled, since it was not designed to isolate the wavelength effect from these confounding acquisition and processing differences.

### 5.1. LOS Displacement and Precipitation

The spatial patterns of deformation velocity derived from both sensors demonstrate that multi-temporal InSAR can capture the extent of slow-moving landslides in tropical mountainous environments. In both study areas, the main deformation zones identified through Sentinel-1 and SAOCOM-1 are consistent with the mapped landslide extents and field observations. However, the two sites differ in the setting of the movement, the magnitude, and in the degree of agreement between the displacement areas and the mapped surface evidence. Sentinel-1 identifies zones of deformation, but the results are more scattered. SAOCOM-1 provides a more spatially coherent representation of the landslide extensions, and this continuity is most evident along the central and downslope portions of both movements.

This contrast is consistent with recent C/L-band comparisons carried out in similarly steep and vegetated terrain. A study conducted in the mountainous catchments of Mao County [4] reported a measurement-point density for L-band ALOS-2 sixteen times higher than that obtained from C-band Sentinel-1 over the same slopes, attributing this difference to both the finer native resolution of ALOS-2 and the weaker canopy penetration capability of C-band wavelengths. A related limitation was reported in [52] for a Sentinel-1-based regional landslide inventory, where the medium ground resolution of C-band data (∼10 m) constrained the ability to resolve internal landslide kinematics, particularly for medium- and small-sized landslides. This limitation is consistent with the more fragmented and less spatially detailed Sentinel-1 deformation signal observed in this study relative to SAOCOM-1.

In Valparaíso, both sensors resolve a well-defined deformation corridor aligned with the slope and coincident with the axis of the mapped movement. This geometry is consistent with the structural setting of the site: the claystones, siltstones and sandstones of the upper member of the Amagá Formation dip towards the northwest and define the structural cuesta north of the urban centre along which the landslide has developed. A translational rupture controlled by bedding along a dip slope is expected to produce displacement that is laterally confined by the strata geometry. The correspondence extends to the direction of motion: the displacement resolved by both sensors is oriented northward, following the same sense in which the strata dip and the slope angle decreases.

The relationship with rainfall at this site appears episodic rather than continuous. The accumulated LOS displacement series show a progressive downslope trend punctuated by inflections that coincide with the rainfall peaks of mid-2024 and early 2025, with cumulative displacements exceeding 65 mm by the end of the monitoring period. This temporal coincidence is consistent with a movement whose reactivation could be associated with short-term pore-water pressure pulses in a low-permeability, clay-rich substrate, where each rainfall episode would produce a discrete acceleration followed by a return to a slower background rate; however, this interpretation remains unverified in the absence of inclinometer or other in-situ deformation data resolving displacement at the daily scale of the rainfall record.

A relevant observation concerns the northern sector of the landslide. There, field evidence is limited to a small number of surface cracks, yet both sensors indicate deformation rates of 40 mm/year. This suggests that the movement could extend beyond the area where surface expression has not been documented and that the mapped extent derived from field inspection may underestimate the true footprint of the instability. For a site where the intermunicipal road to other municipalities is the single access route to the urban center, this discrepancy has direct implications for the delineation of the exposure area.

In Venecia the deformation field is concentrated within the mapped landslide body adjacent to the urban boundary, with the surrounding slopes remaining comparatively stable, and the resolved displacement is oriented northward, in the same orientation as at Valparaíso. Both the lithological and the geomorphological context are shared by the two sites, since both movements developed on the Amagá Formation and on the same type of structural cuesta; the deformation pattern, however, is more compact and less elongated.

The temporal signature also differs. The Sentinel-1 series shows a steady and continuous increase in negative displacement reaching values close to 40 mm, without the discrete inflections observed in Valparaíso, and the rainfall record indicates a marked increase in event frequency during the second half of the observation period, with several peaks exceeding 40–60 mm. These observations are suggestive of a possible response governed by cumulative infiltration and delayed pore-pressure build-up within a thick regolith, in contrast to the more immediate response to individual storms inferred for Valparaíso; this contrast cannot be confirmed in the absence of a dedicated inclinometer or other in-situ deformation measurements.

The northern portion of the Venecia landslide presents the inverse of the situation described at Valparaíso: numerous surface cracks have been reported there, yet neither sensor resolves significant deformation, with only Sentinel-1 indicating localized displacements on the order of 40 mm/year. The absence of a clear InSAR signal does not necessarily imply the absence of ground deformation. The displacement in that sector may be oriented close to perpendicular to the descending line of sight, in which case the projected component of a real displacement would fall below the detection threshold. Also, the age of the observed cracks is uncertain; some may reflect inactive or displacements from earlier deformation episodes rather than active movement during the observation period, which would explain their presence without a corresponding contemporary InSAR signal.

### 5.2. Vegetation Cover and Interferometric Coherence

In the Valparaíso study area, the Sentinel-1 distribution reflects the influence of dense vegetation cover and the environmental conditions typical of tropical mountainous regions, which degrade the phase stability of C-band signals. This behavior is supported quantitatively: 60.63% of the valid Sentinel-1 pixels reached coherence values equal to or greater than the adopted threshold of 0.4, with a mean of 0.43 and a median of 0.41, whereas 99.43% of the valid SAOCOM-1 pixels exceeded the same threshold, with a mean of 0.69 and a median of 0.71. In the Venecia study area, the same contrast is observed at a higher overall level, with 81.47% of Sentinel-1 pixels and 98.72% of SAOCOM-1 pixels above the threshold, and medians of 0.47 and 0.75, respectively. The SAOCOM-1 distributions remain high above the threshold at both sites, indicating improved phase stability and stronger interferometric performance over the same areas.

The stratification by land cover shows that the two sensors diverge most precisely over the vegetated categories that dominate both study areas. Over urban surfaces the two bands are close, with median coherences of 0.76 and 0.85 at Venecia and 0.63 and 0.72 at Valparaíso, a gap of about 0.09 at both sites. Over sparse vegetation and dense forest, which together account for 72.5% of the Venecia map and 86.8% of the Valparaíso map, the gap widens to between 0.24 and 0.31: Sentinel-1 medians fall to 0.45 and 0.53 at Venecia and to 0.41 and 0.41 at Valparaíso, whereas SAOCOM-1 retains values close to 0.71 and 0.77 at Venecia and 0.71 and 0.72 at Valparaíso. This difference in coherence level translates directly into the quality of the phase measurement. The variance of the interferometric phase is inversely related to coherence, so that a pixel with a coherence of 0.7 carries a phase estimate whose dispersion is substantially smaller than that of a pixel at 0.45, and the propagation of that dispersion through the network inversion determines the precision of the retrieved displacement. Over sparse vegetation and dense forest, the Sentinel-1 medians lie only marginally above the 0.4 masking threshold, which means that even the pixels retained for interpretation are drawn from a regime where the phase is close to the limit of usability. The SAOCOM-1 distributions over the same categories are centered well above that regime and are narrow, indicating that the phase quality is not only higher on average but also more uniform across the vegetated terrain. Displacement estimates obtained from L-band over these covers are therefore less noisy. Since vegetated categories constitute most of the area of both landslide extensions, this advantage applies precisely to the surfaces where the deformation signal has to be recovered. A final aspect to consider is the land cover characterization, which relies on unsupervised classification. This approach allows the vegetation conditions to be described consistently across both sites, but no accuracy assessment was performed, so the resulting categories should be understood as spectral classes. The four categories correspond to the dominant land cover types of the Andean landscape and are consistent with the classes reported for the study areas by the ESA WorldCover product [62], which at 10 m resolution identifies the same categories: urban (built-up), grassland, sparse vegetation, and forest (tree cover). Extending the analysis with a supervised classification trained on photo-interpreted samples is identified here as a direction for future work.

This pattern closely reproduces the conclusions reported in [4] from a direct comparison of Sentinel-1 and the L-band ALOS-2 mission over steep, vegetated slopes in southwest China: L-band consistently outperformed C-band in coherence, observation coverage, and the magnitude of recoverable displacement, while C-band remained better suited to detecting comparatively slight movements where coherence was preserved. The quantitative results obtained in this study reinforce this interpretation, since the fraction of coherent pixels above the adopted threshold was substantially greater for SAOCOM-1 than for Sentinel-1 in both study areas. Because SAOCOM-1 operates at a similar L-band wavelength (∼23.5 cm) to ALOS-2, the convergence between these two independent case studies—one in subtropical China and the other in the tropical northern Andes—suggests that the coherence advantage of L-band over C-band in vegetated, steep terrain is governed primarily by wavelength-dependent canopy penetration rather than by site-specific conditions. The same physical control has been documented under markedly different climatic conditions in [61], where Sentinel-1 coherence over a boreal forest catchment decreased to 0.2–0.3 during the leaf-on, high-moisture summer months and recovered to 0.5–0.6 once vegetation and surface-moisture activity subsided in winter. These observations indicate that vegetation- and moisture-driven decorrelation is a limitation of C-band InSAR across different climatic environments.

However, this coherence contrast was not obtained under identical acquisition and processing conditions. Sentinel-1 and SAOCOM-1 differ in revisit interval (6 days effective in this study and 8 days for SAOCOM-1, combining both constellation satellites), incidence-angle range, temporal and spatial baseline thresholds used to build the interferometric network (≤60days/180m compared with ≤180days/2000m), multilooking factor, resulting in effective pixel sizes of 32 m and 48 m, respectively, and processing software, all of which can influence coherence estimation independently of wavelength. Notably, the SAOCOM-1 network was built using considerably longer temporal and spatial baselines than Sentinel-1, a configuration that would ordinarily be expected to increase, rather than decrease, decorrelation. The fact that SAOCOM-1 nonetheless retained higher and more spatially consistent coherence despite these comparatively unfavorable baseline conditions suggests that the observed advantage is unlikely to be fully explained by network configuration alone, lending support to a genuine wavelength-dependent effect. Even so, the coarser effective pixel size of SAOCOM-1 also increases the number of looks used in coherence estimation, which independently tends to raise and smooth apparent coherence values, so this factor cannot be entirely disentangled from the wavelength effect with the present processing setup. A fully controlled comparison—using matched temporal baselines, equivalent coherence-estimation windows, and a common processing framework for both sensors—would be required to isolate the wavelength contribution more rigorously, and is identified here as a direction for future work.

These results point to two complementary paths for improving future monitoring in similar tropical catchments. First, the seasonal coherence-optimization strategy demonstrated in [61]—restricting the interferometric stack to the time of year with the most favorable scattering conditions, rather than processing all acquisitions uniformly—could be adapted to the Andean setting by preferentially weighting interferometric pairs from comparatively drier periods, potentially mitigating part of the C-band decorrelation documented here for Sentinel-1. Second, the persistent resolution and coherence gap between Sentinel-1 and longer-wavelength sensors echoes the outlook presented in [52], which anticipates that forthcoming longer-wavelength, higher-resolution, short-revisit missions (e.g., LT-1, Tandem-L) will be needed to close this gap for regional-scale early detection. In the meantime, the combined use of a C-band mission for broad temporal coverage and an L-band mission such as SAOCOM-1 for spatial coherence is a practical strategy for landslide characterization in densely vegetated, steep tropical terrain.

### 5.3. Geometric Distortion

Geometric distortions are an important limitation of SAR observations in mountainous terrain, where the interaction between the radar viewing geometry and the local topography may produce foreshortening, layover, or shadow [4]. Their occurrence is controlled primarily by the relationship between the local slope, terrain aspect, radar incidence angle, and sensor look direction. Slopes facing the radar may undergo foreshortening when the terrain inclination approaches the local incidence geometry, whereas steeper configurations may lead to layover. Conversely, slopes facing away from the sensor may progressively lose radar illumination and eventually become affected by shadow. Therefore, the spatial distribution of these distortions depends on the specific acquisition geometry of each sensor and on its interaction with the terrain, rather than on radar wavelength alone.

The results presented in Figure 15 and Figure 16 illustrate this dependence and show clear differences between the two study areas. Foreshortening is the dominant distortion class in both Venecia and Valparaíso, whereas layover and shadow account for only a negligible proportion of the analyzed areas. This pattern is consistent with the predominantly moderate-to-steep slopes facing the radar in both study sites, where terrain compression is more common than the extreme geometric configurations required to generate layover or complete radar shadow.

In Venecia, both sensors show very similar geometric responses. Suitable-observation terrain represents 75% of the total study area and increases to about 93–94% within the mapped landslide body. The landslide boundary and most of the reported surface cracks are therefore located within geometrically favorable sectors for both Sentinel-1 and SAOCOM-1, with only a narrow portion affected by foreshortening. Under these comparatively gentler terrain conditions, the differences between the acquisition geometries of the two sensors have only a limited influence on the spatial reliability of the observations.

A different behavior is observed in Valparaíso, where the elongated landslide corridor is located on steeper and more uniformly inclined terrain. In this case, SAOCOM-1 provides suitable-observation conditions over 64% of the landslide body, compared with 51% for Sentinel-1, while the corresponding foreshortening fractions are about 35% and 48%, respectively. As shown in Figure 15c,d, this difference is particularly relevant in the upper and central portions of the landslide, where numerous surface cracks have been reported. Consequently, the SAOCOM-1 acquisition geometry provides more favorable geometric conditions over a larger fraction of the field-confirmed unstable area, reducing the proportion of the landslide affected by terrain compression relative to Sentinel-1.

These results indicate that the effect of acquisition geometry becomes increasingly important as terrain steepness and slope orientation become less favorable for radar observation. In Venecia, where topographic conditions are comparatively less restrictive, both sensors provide nearly equivalent geometric coverage. In Valparaíso, however, differences in sensor viewing geometry substantially modify the proportion of the landslide that can be interpreted with greater geometric confidence. This effect is independent of, but complementary to, the wavelength-related differences in interferometric coherence discussed in Section 4.2: coherence controls the temporal stability of the phase signal, whereas geometric distortions determine whether the terrain is favorably represented by the radar acquisition geometry.

The comparison must nevertheless be interpreted considering the different effective pixel sizes of the two processed datasets. After multilooking, the SAOCOM-1 products have an effective pixel size of 48 m, compared with 32 m for Sentinel-1. Because the distortion classification was performed on a per-pixel basis, the greater spatial aggregation of the SAOCOM-1 product may smooth local topographic variability and modify the apparent proportions assigned to each geometric class. Therefore, the differences in suitable-observation and foreshortening percentages cannot be attributed exclusively to acquisition geometry, and part of the observed contrast may also reflect the different spatial resolutions of the processed products.

### 5.4. Limitations

A significant limitation concerns validation. The field information available for both sites consists of landslide extension mapping and identification of surface cracks, which establish that instability is present but do not measure its rate. No inclinometer, extensometer, or GNSS record was available for either study area during the observation period, so the InSAR displacement magnitudes reported should be treated as estimations of the displacement rather than as validated velocities.

A second limitation concerns the comparability of the two datasets themselves. Beyond the difference in radar wavelength, Sentinel-1 and SAOCOM-1 differ in acquisition mode and incidence-angle range, number of available scenes, interferometric network configuration, effective pixel size after multilooking, and processing software. These differences arise partly from data-availability constraints, Sentinel-1 SLC data are readily accessible through ASF, whereas SAOCOM-1 data require a separate acquisition and processing route, rather than from a deliberate attempt to match the two pipelines. As a result, the comparison presented here cannot fully isolate the effect of wavelength from these other sensor- and processing-specific factors, and the observed differences in coherence, spatial continuity, and detectable area should be interpreted as reflecting the combined influence of all these variables rather than the radar band alone.

A third set of limitations affects the interpretation of the deformation time series itself. The observation window spans 19 months and therefore covers only three rainy seasons, which could be insufficient to establish a correspondence between rainfall and displacement and to compare the influence of individual events against interannual modulation associated with ENSO. Future work should also include the installation of ground-based instrumentation: inclinometers to constrain the depth of the failure surface, and piezometers to measure the pore-pressure response that is here inferred solely from rainfall. The introduction of in-situ deformation sensors would allow the rainfall–displacement relationship to be expressed as a physical model rather than as a temporal correspondence.

Finally, the approach applied here to two individual slopes should be extended to the regional scale. The southwestern Antioquia region concentrates a substantial number of slow-moving landslides in comparable lithological and climatic settings, and a systematic multi-frequency inventory would allow the site-specific observations reported in this study to be tested for generality and would provide the deformation baseline required for integrating InSAR into the operational landslide early warning framework currently being developed for the region.

## 6. Conclusions

This study applied SBAS-InSAR techniques to identify and characterize slow-moving landslides in the northern tropical Andes using Sentinel-1 (C-band) and SAOCOM-1 (L-band) SAR datasets. The InSAR-derived deformation patterns were contrasted with independent field evidence, including mapped landslide extents and reported surface cracks, which supported the spatial interpretation of the detected movements but did not constitute a systematic ground-based validation of displacement rates. In addition, the study compared interferometric coherence, land cover, displacement evolution, precipitation, and geometric distortions for both sensors. The main conclusions are summarized as follows:

First, both Sentinel-1 and SAOCOM-1 successfully identified deformation patterns spatially consistent with the mapped landslides in Venecia and Valparaíso, confirming the applicability of multi-temporal InSAR for detecting slow-moving slope instability in tropical mountainous environments. Deformation rates were on the order of several tens of millimeters per year in both study areas. However, SAOCOM-1 generally provided a more spatially continuous representation of the deformation field, particularly over vegetated terrain, whereas the Sentinel-1 results were comparatively more fragmented.

Second, some of the negative displacement increments in both study areas coincide with days of markedly higher precipitation than the surrounding period, which could be compatible with a response governed by infiltration, although in-situ instrumentation would be required to confirm such an acceleration of the mechanism. In addition, the slow-moving landslides correspond to weathered soils overlying an inherited and irregular surface developed on the sedimentary rocks of the Amagá Formation, a configuration attributed to the propagation of older landslides from the steep volcanic topography of the Combia Formation onto the subdued relief of the sedimentary sequence [43]. This landscape evolution would have reorganized the drainage network and the preferential paths of water circulation, so that infiltrated water accumulates in the depressions of the buried surface, progressively reducing the effective stress favoring displacement along the direction of dip of that surface. The consistent northward orientation of both movements is coincident with the dip of the strata and with the direction in which the slope angle decreases. Nevertheless, the mechanism cannot be verified with the available observations.

Third, the coherence analysis revealed a consistent contrast between the C-band and L-band datasets. Over the total study areas, SAOCOM-1 retained 99.43% and 98.72% of valid pixels above the adopted coherence threshold of 0.4 in Valparaíso and Venecia, respectively, compared with 60.63% and 81.47% for Sentinel-1. The corresponding median coherence values were also higher for SAOCOM-1 (0.714 in Valparaíso and 0.751 in Venecia) than for Sentinel-1 (0.415 and 0.478, respectively). The separation between sensors was greatest over sparse vegetation and dense forest, which dominate both study areas, while the differences were smaller over urban surfaces. These results quantitatively support the greater phase stability of the L-band observations under vegetated conditions and explain, at least in part, the greater spatial continuity of the SAOCOM-1 deformation products.

Fourth, the geometric distortion analysis showed that foreshortening was the dominant distortion class in both study areas, whereas layover and shadow affected only a small proportion of the analyzed terrain. The influence of geometric distortions differed substantially between the two sites. In Venecia, both sensors provided nearly equivalent geometric coverage, with 93–94% of the mapped landslide body classified as suitable for observation. In Valparaíso, where the landslide occupies steeper and more uniformly inclined terrain, SAOCOM-1 preserved suitable-observation conditions over 64% of the landslide body, compared with 51% for Sentinel-1, while foreshortening affected 35% and 48%, respectively. These results indicate that the geometric observability of a landslide depends primarily on the interaction between terrain orientation and the specific viewing geometry of each sensor, including incidence angle and look direction, rather than on radar wavelength itself. Accordingly, the higher coherence observed for SAOCOM-1 and its more favorable geometric coverage in Valparaíso represent two distinct but complementary effects: wavelength influences the preservation of interferometric phase over vegetation, whereas acquisition geometry controls the susceptibility of the terrain to foreshortening, layover, and shadow.

The comparison should nevertheless be interpreted considering the differences between the two datasets. Sentinel-1 and SAOCOM-1 differ in acquisition mode, incidence-angle range, number of available scenes, interferometric-network configuration, effective pixel size after multilooking, and processing workflow. Consequently, the observed differences cannot be attributed exclusively to radar wavelength. In particular, the coarser effective pixel size of the SAOCOM-1 products may contribute to smoother coherence fields and may also influence the apparent proportions of geometric distortion classes.

The results demonstrate the complementary value of C- and L-band observations for monitoring slow-moving landslides in tropical mountainous environments. Sentinel-1 provides dense temporal sampling and broad operational availability, while SAOCOM-1 offers greater coherence preservation and, in the study areas analyzed here, more spatially continuous deformation information over vegetated terrain. Their combined use therefore provides a more robust basis for landslide detection and characterization than either dataset alone, particularly where dense vegetation and complex topography simultaneously constrain InSAR performance.

## Figures and Tables

**Figure 1:**
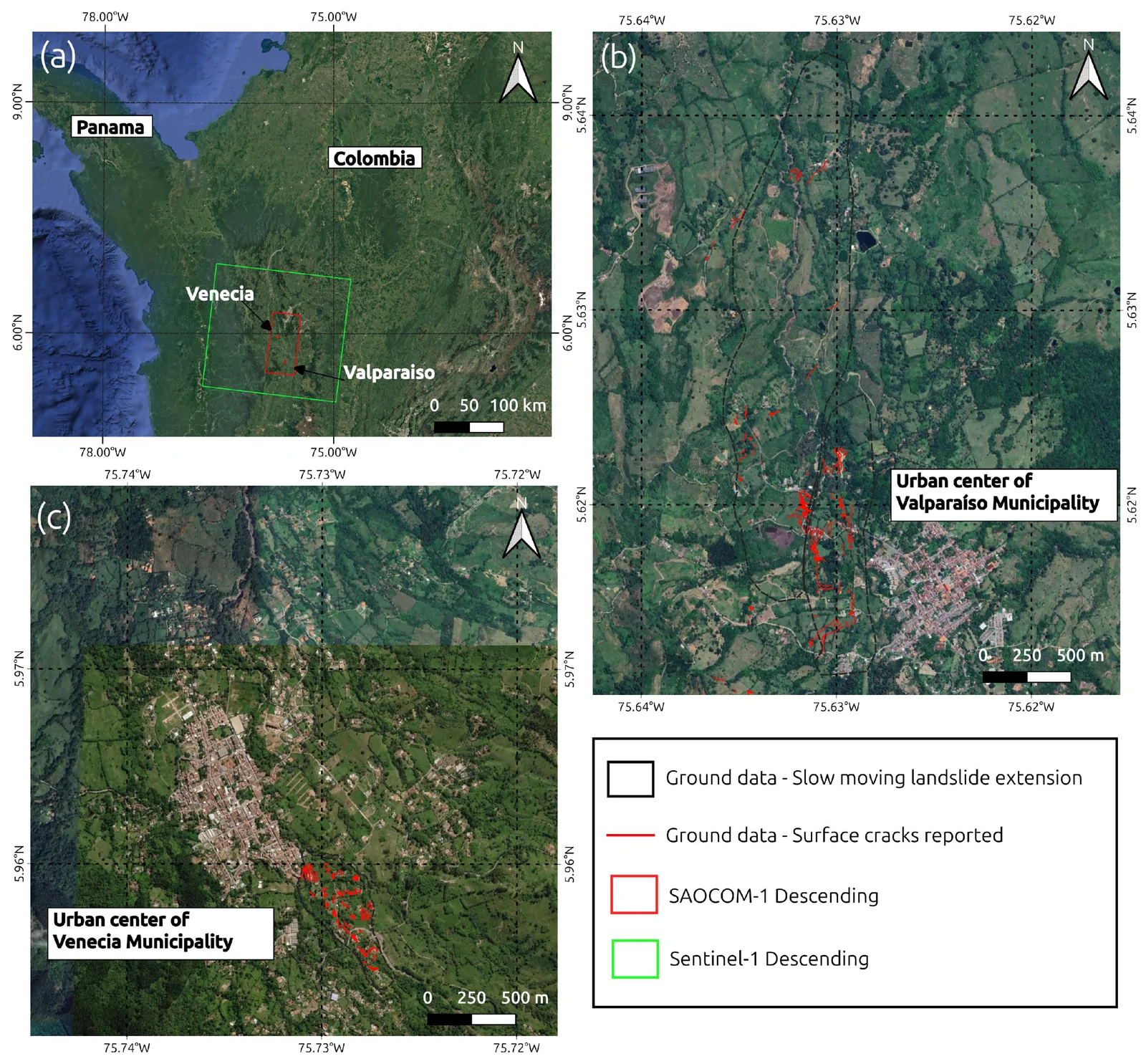
Study area location in southwestern Antioquia, Colombia. (**a**) Regional context map showing the geographic location of both study areas within Colombia. (**b**) Aerial view of the Valparaíso Municipality urban center, showing the mapped slow-moving landslide extent and reported surface cracks. (**c**) Aerial view of the Venecia Municipality urban center, with the corresponding landslide boundary and surface cracks.

**Figure 2:**
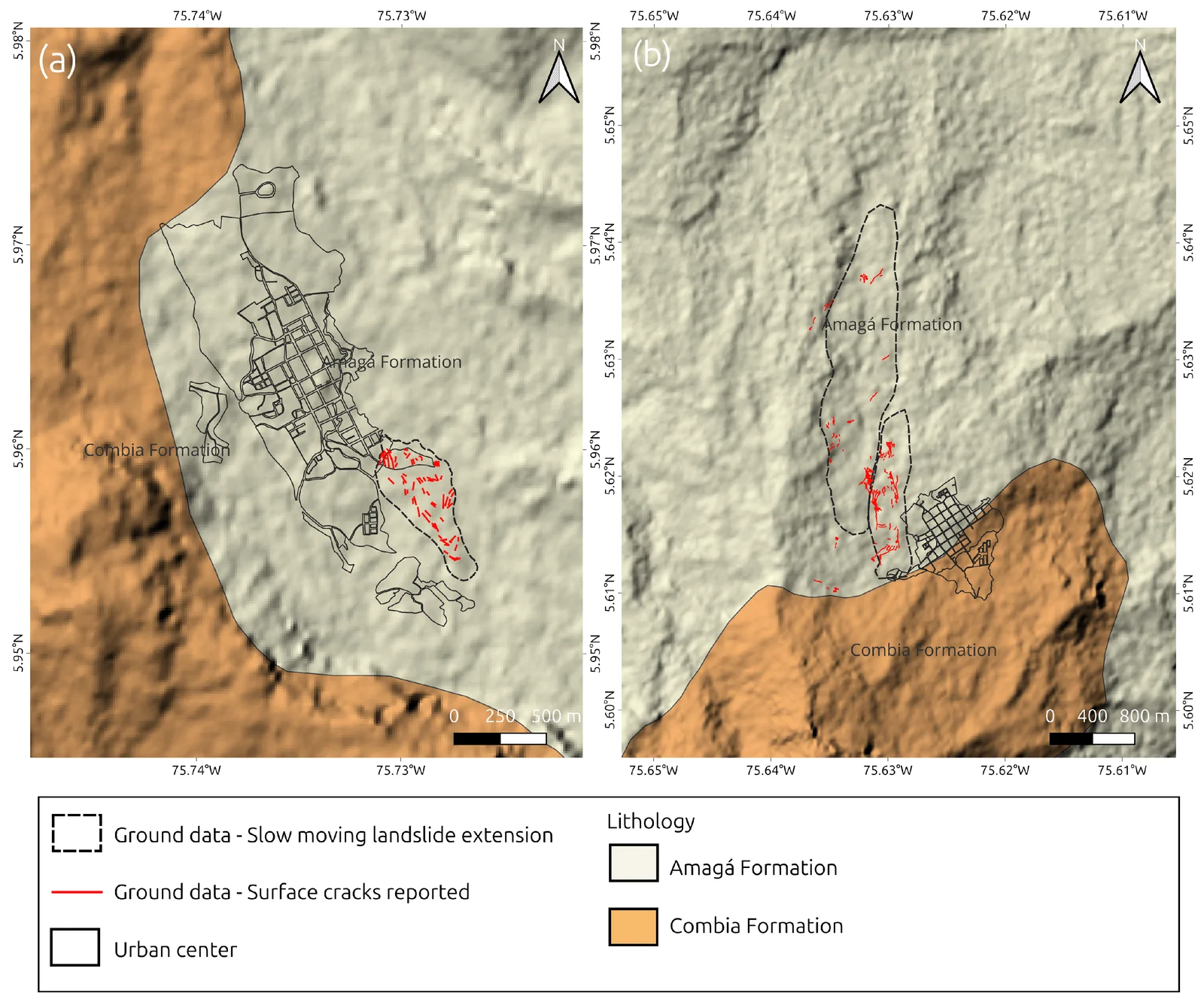
Geological map. (**a**) Venecia study area. (**b**) Valparaiso study area.

**Figure 3:**
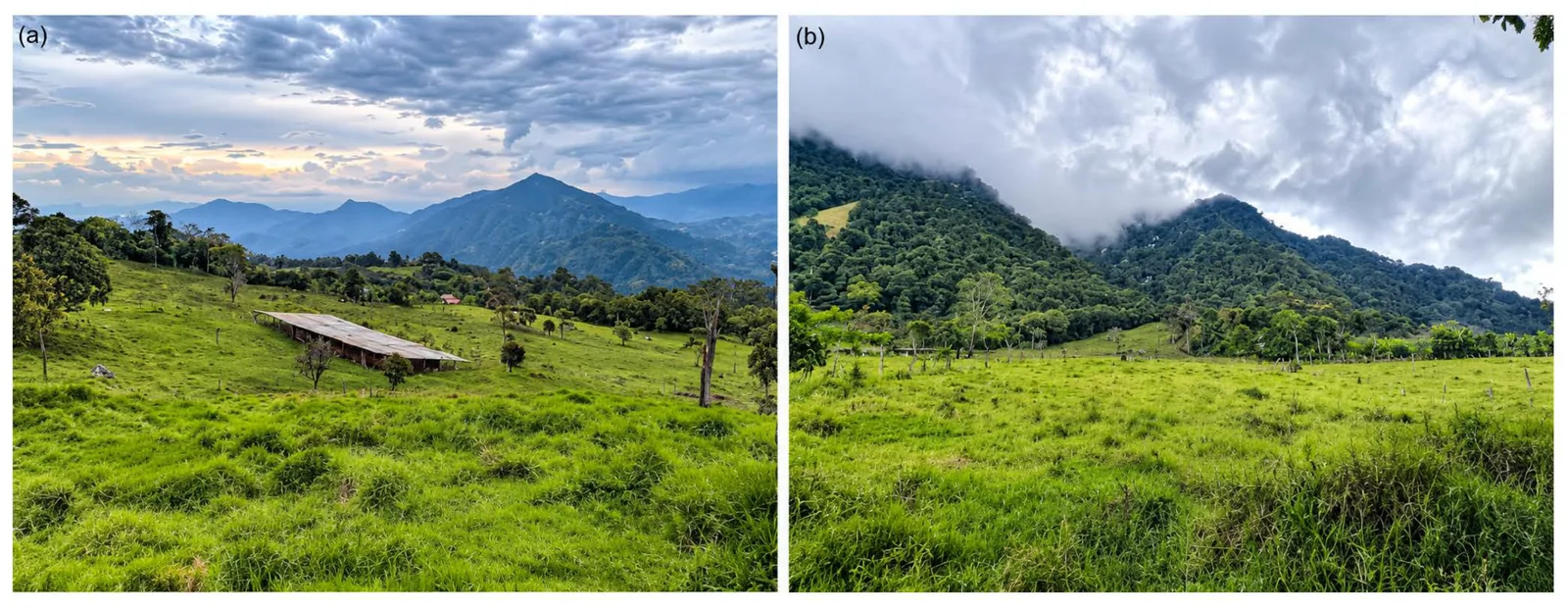
Terrain morphology developed on the Amagá Formation in the Venecia study area. (**a**) Structural cuesta on clastic sedimentary rocks, showing the dip slope. (**b**) Low-gradient sector with smoothed morphology, bounded by steeper slopes on the Combia Formation porphyries.

**Figure 4:**
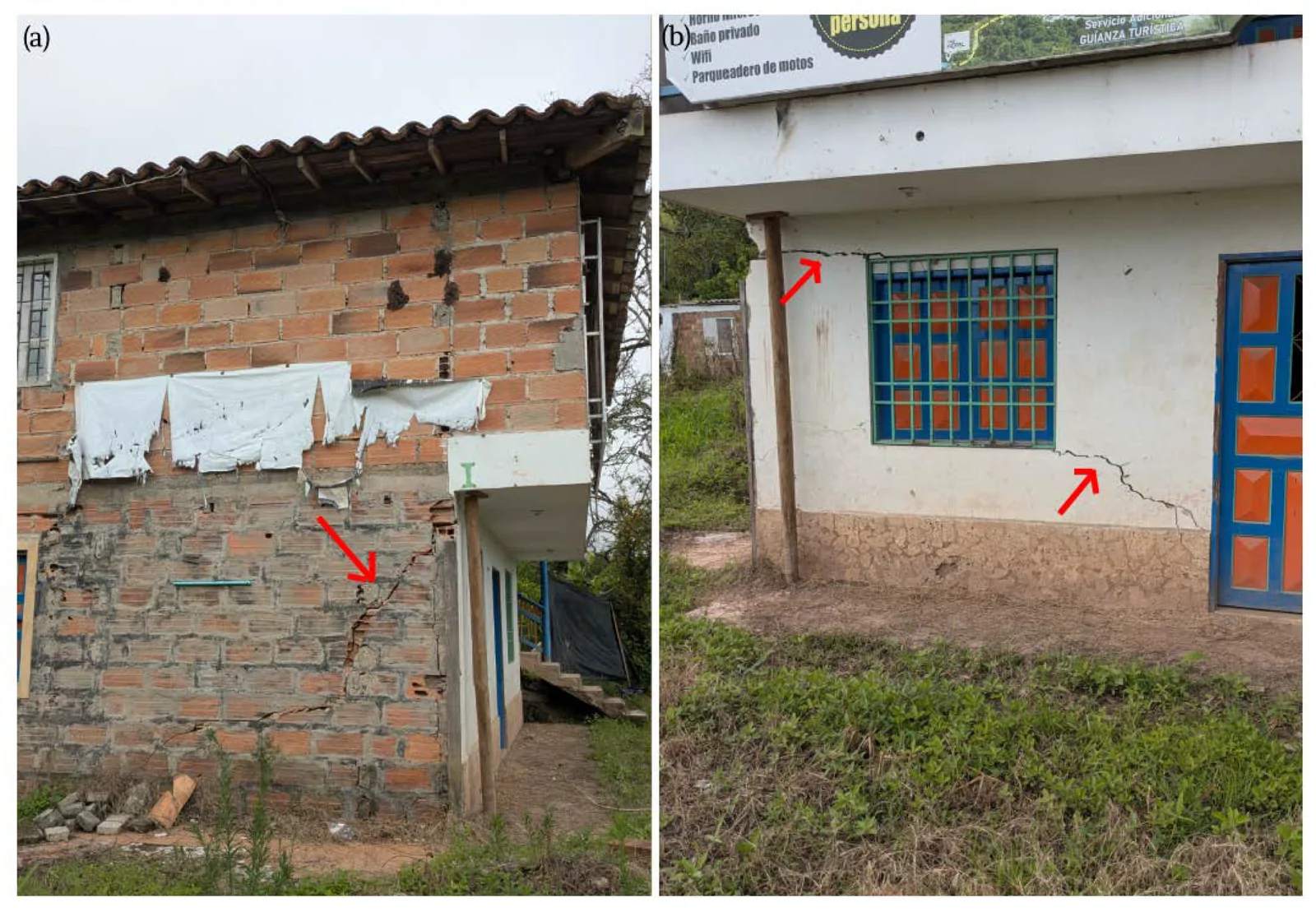
Ground evidence of landslide-related surface deformation in the Valparaíso study area: (**a**,**b**) Surface cracks and ground displacement affecting residential areas.

**Figure 5:**
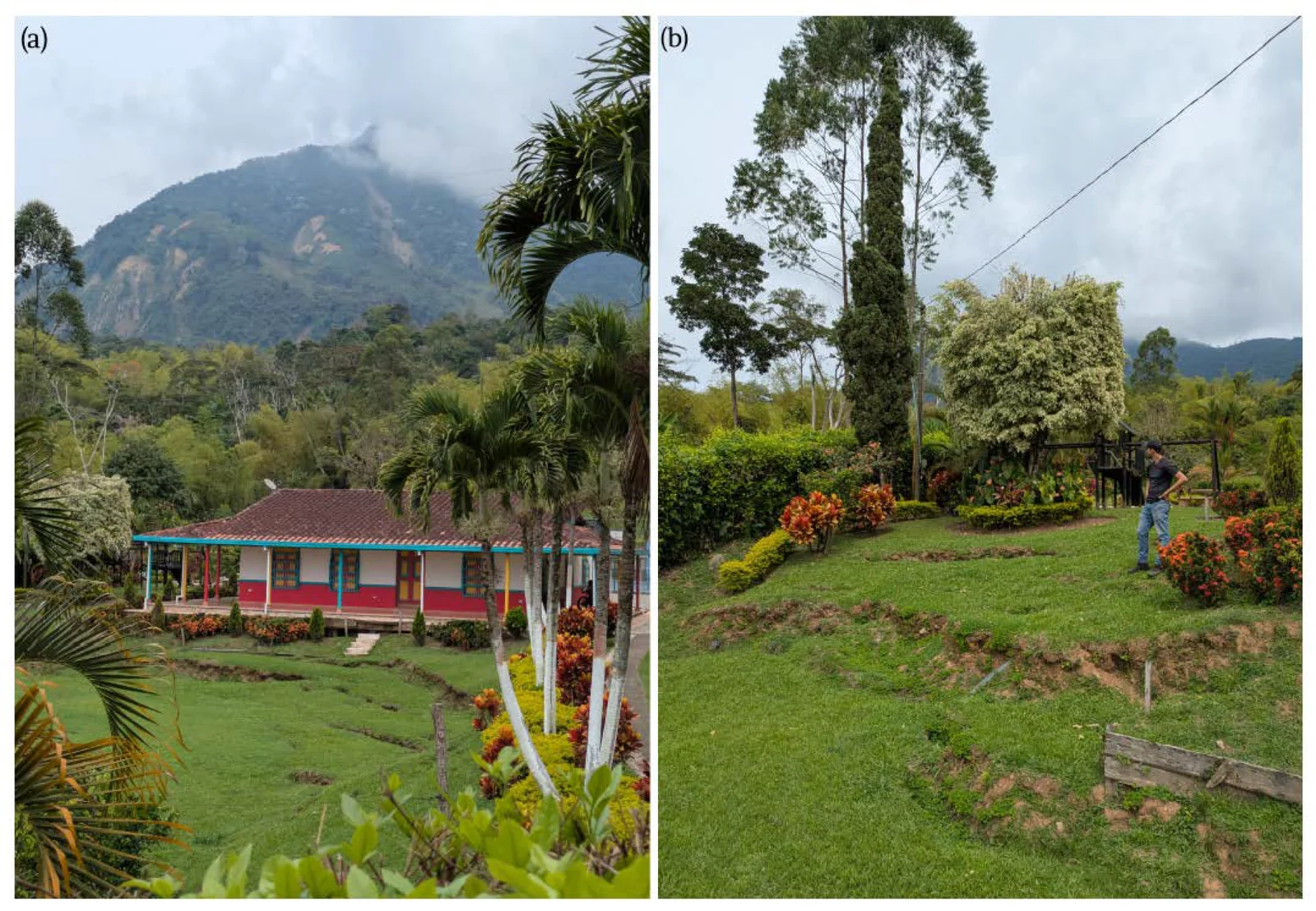
Ground damage associated with the slow-moving landslide in the Venecia study area: (**a**,**b**) Surface cracks and ground displacement affecting residential areas are visible, including soil fissures surrounding houses and structural damage to a masonry wall caused by progressive slope deformation.

**Figure 6:**
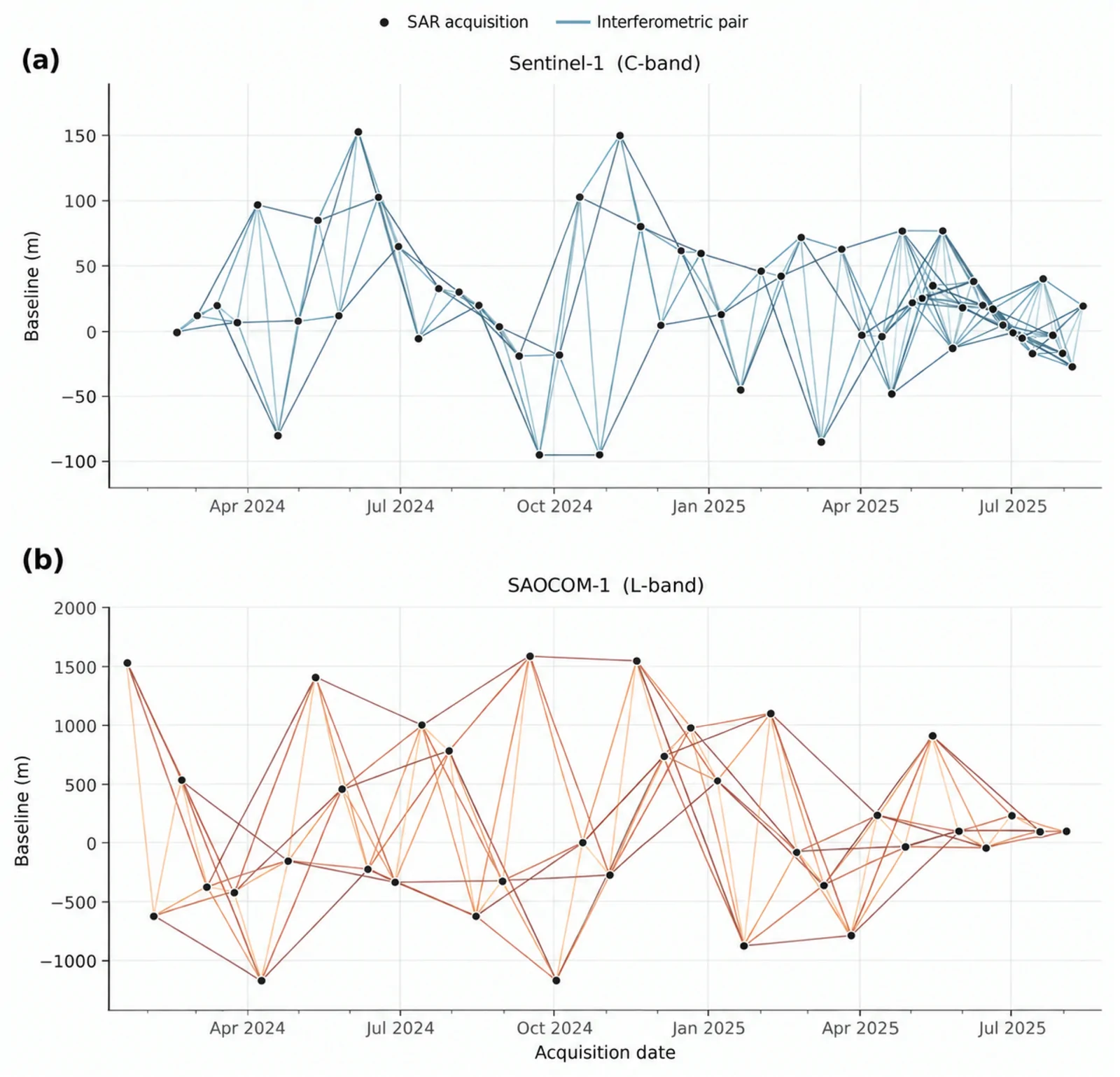
Time-position plots: (**a**) Sentinel-1 descending; (**b**) SAOCOM-1 descending. The vertical axis represents the spatial baseline (in meters), while the horizontal axis indicates the time (in years) of each acquisition. Each point in the figure corresponds to a SAR image, and the lines connecting them represent the interferograms generated between image pairs.

**Figure 7:**
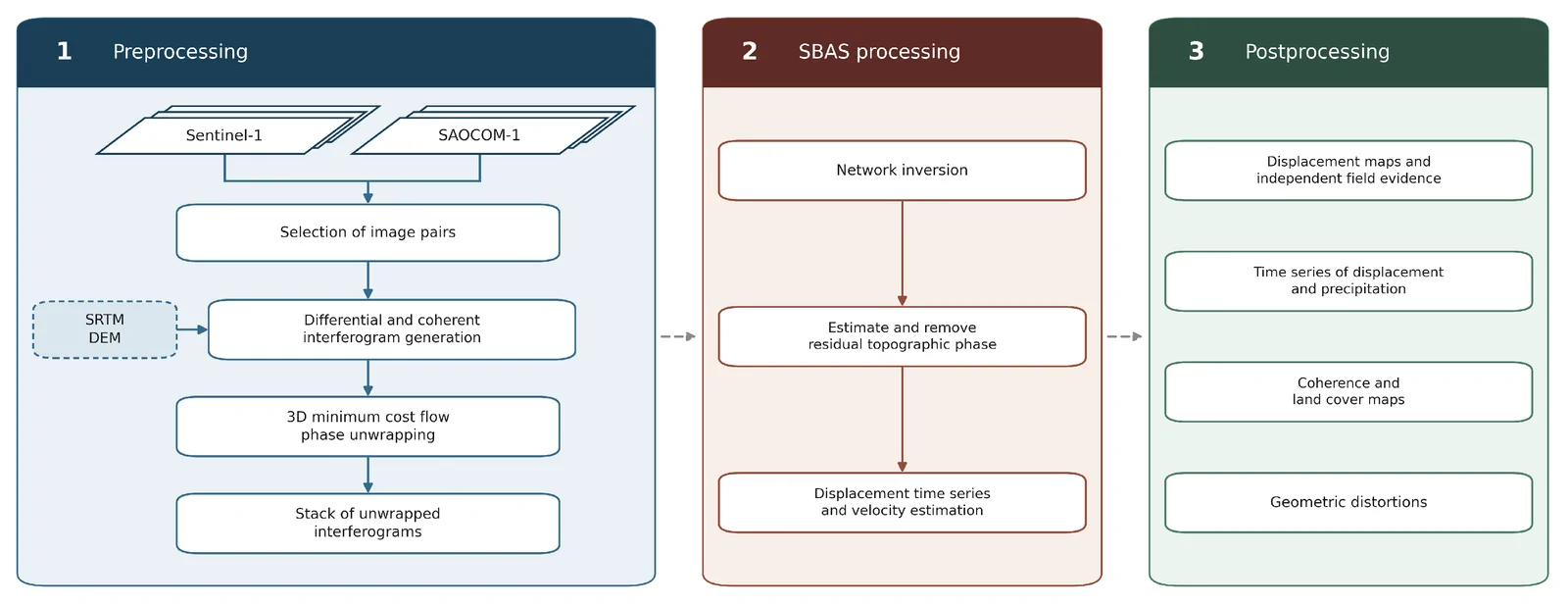
Workflow overview.

**Figure 8:**
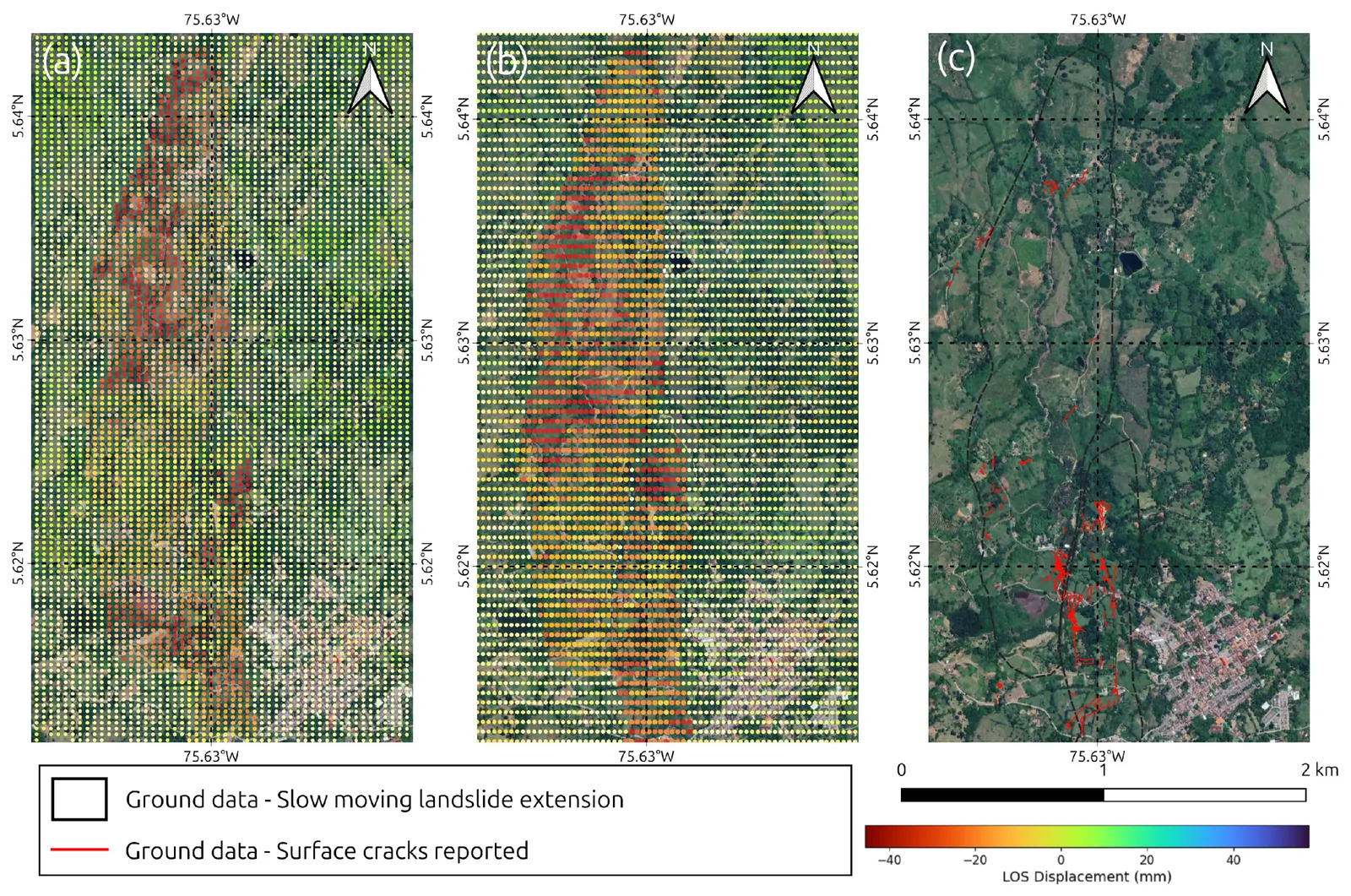
InSAR-derived LOS displacement maps and ground evidence for the Valparaiso study area. (**a**) Sentinel-1 accumulated LOS displacement. (**b**) SAOCOM-1 accumulated LOS displacement. (**c**) Field-validated ground evidence, including mapped slow-moving landslide extent and reported surface cracks.

**Figure 9:**
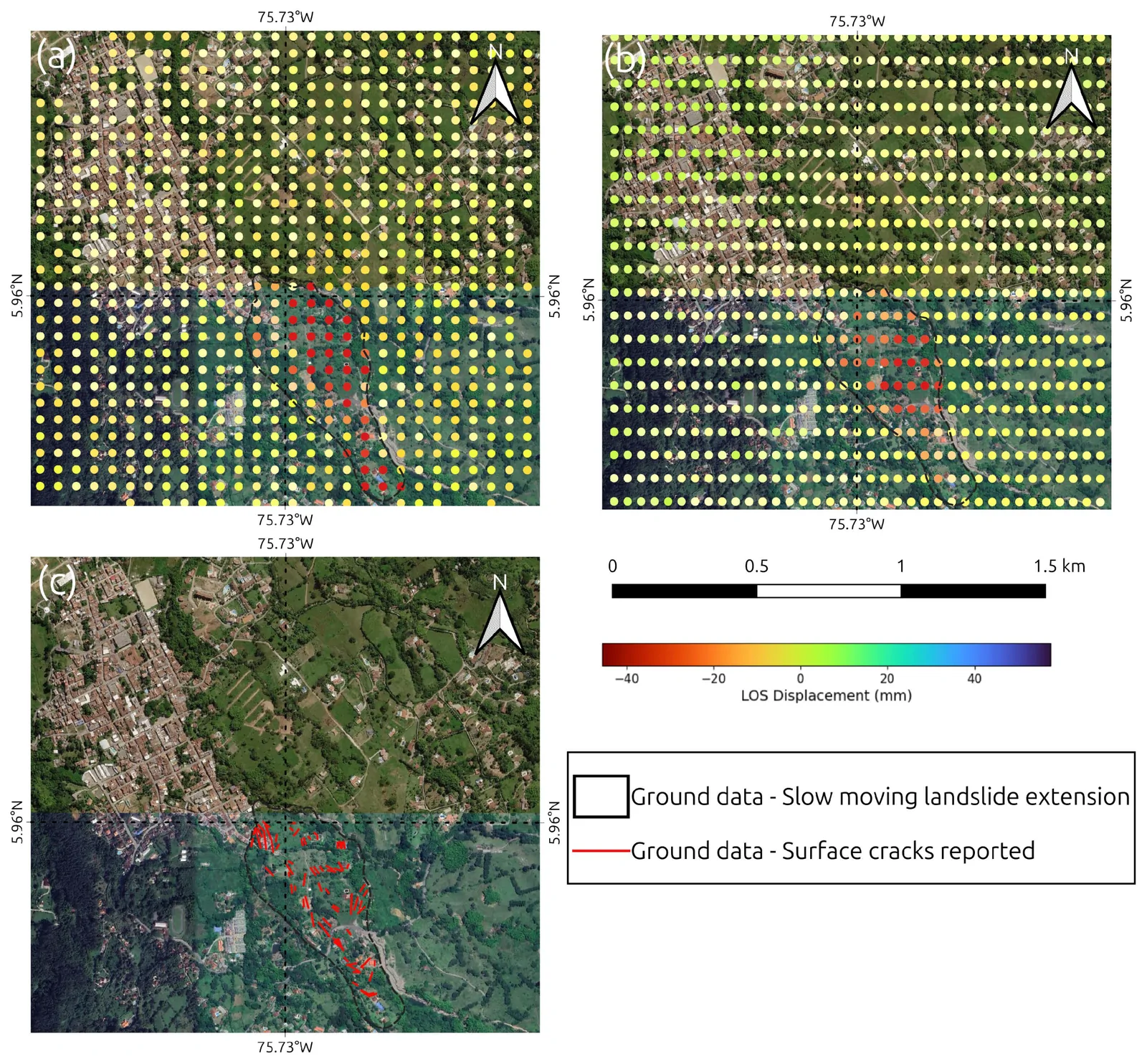
InSAR-derived LOS displacement maps and ground evidence for the Venecia study area. (**a**) Sentinel-1 accumulated LOS displacement. (**b**) SAOCOM-1 accumulated LOS displacement. (**c**) Field-validated ground evidence, including mapped slow-moving landslide extent and reported surface cracks.

**Figure 10:**
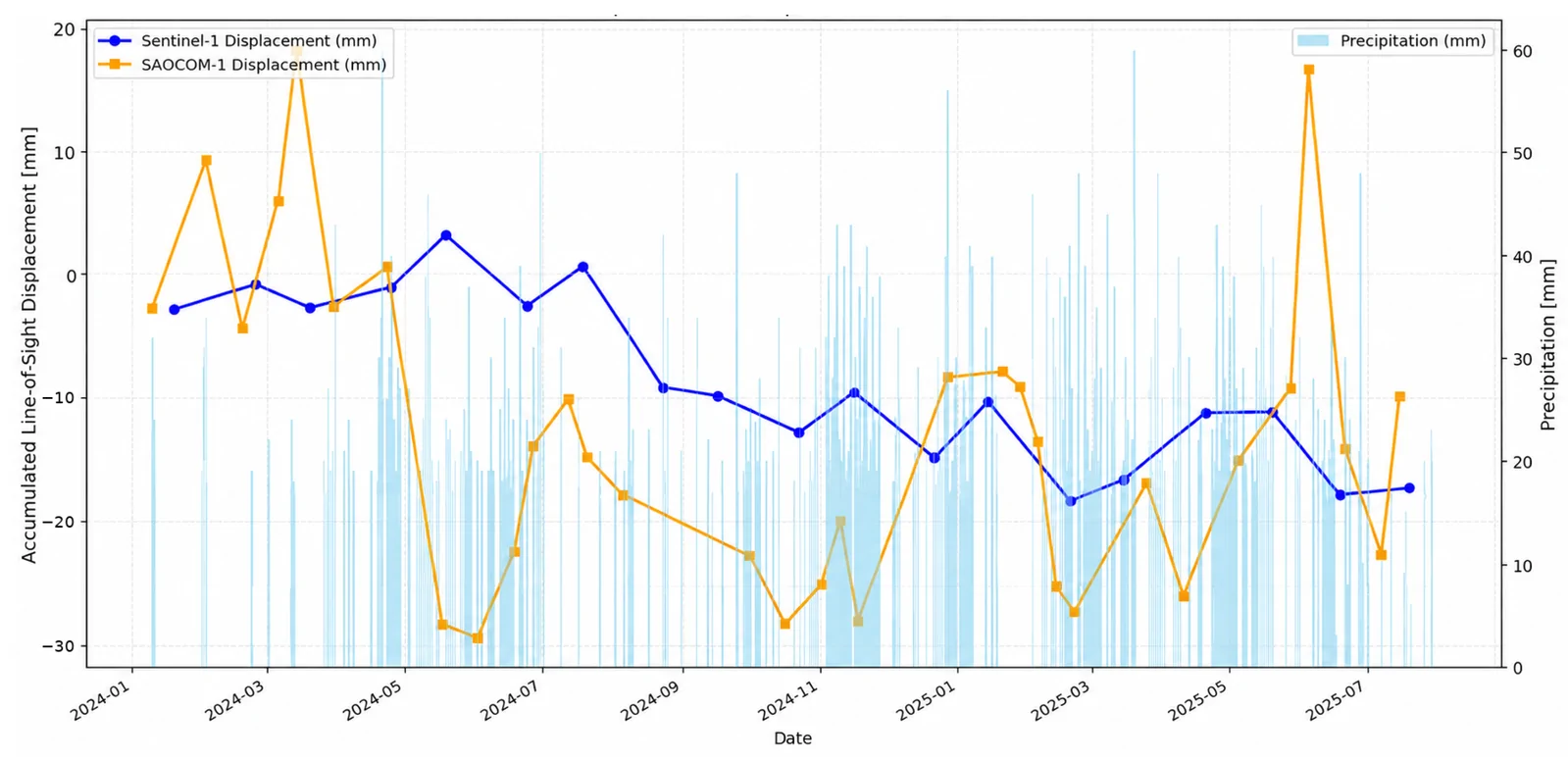
Time series of accumulated InSAR LOS displacement and daily precipitation for the Valparaiso study site, covering January 2024 to August 2025.

**Figure 11:**
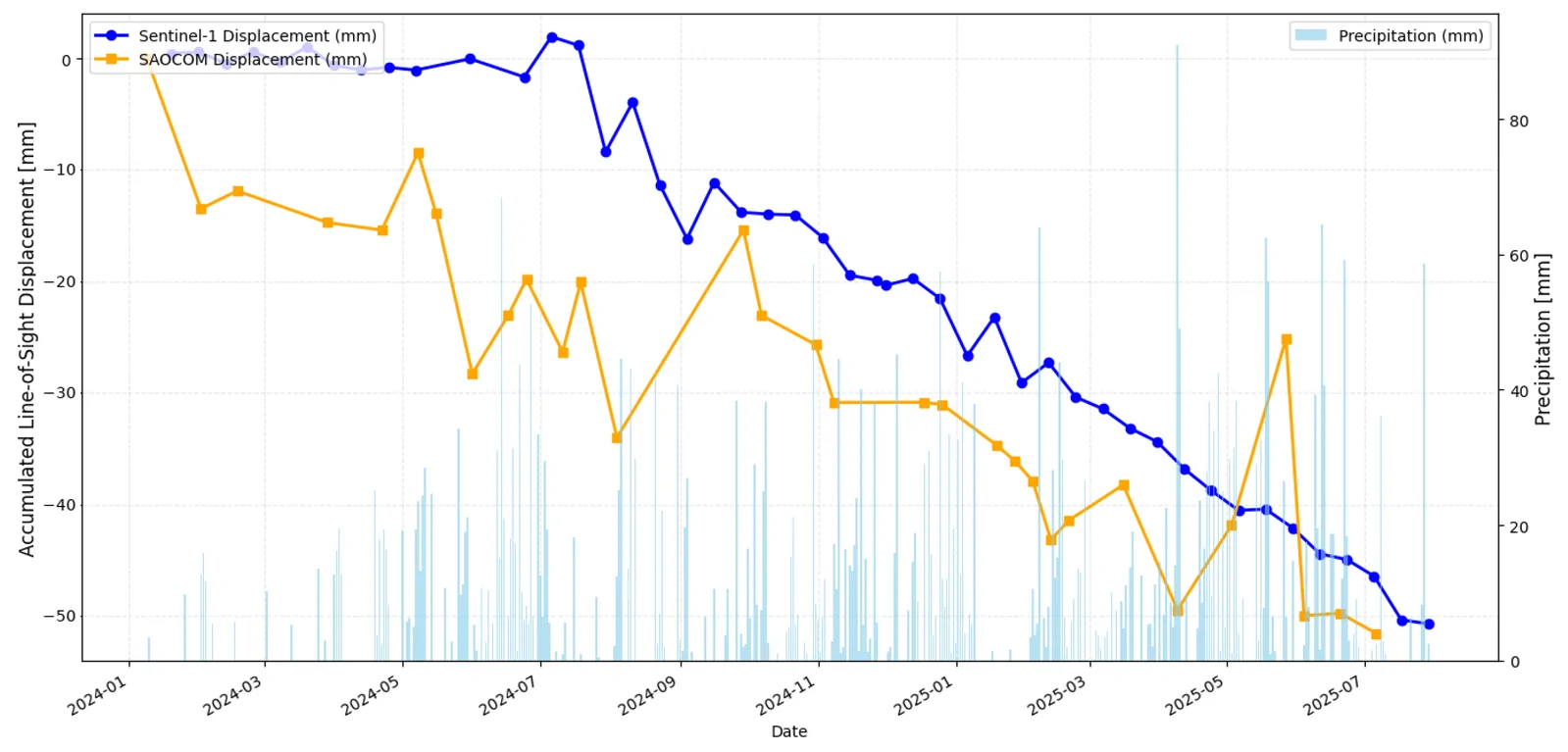
Time series of accumulated InSAR LOS displacement and daily precipitation for the Venecia study site, covering January 2024 to August 2025.

**Figure 12:**
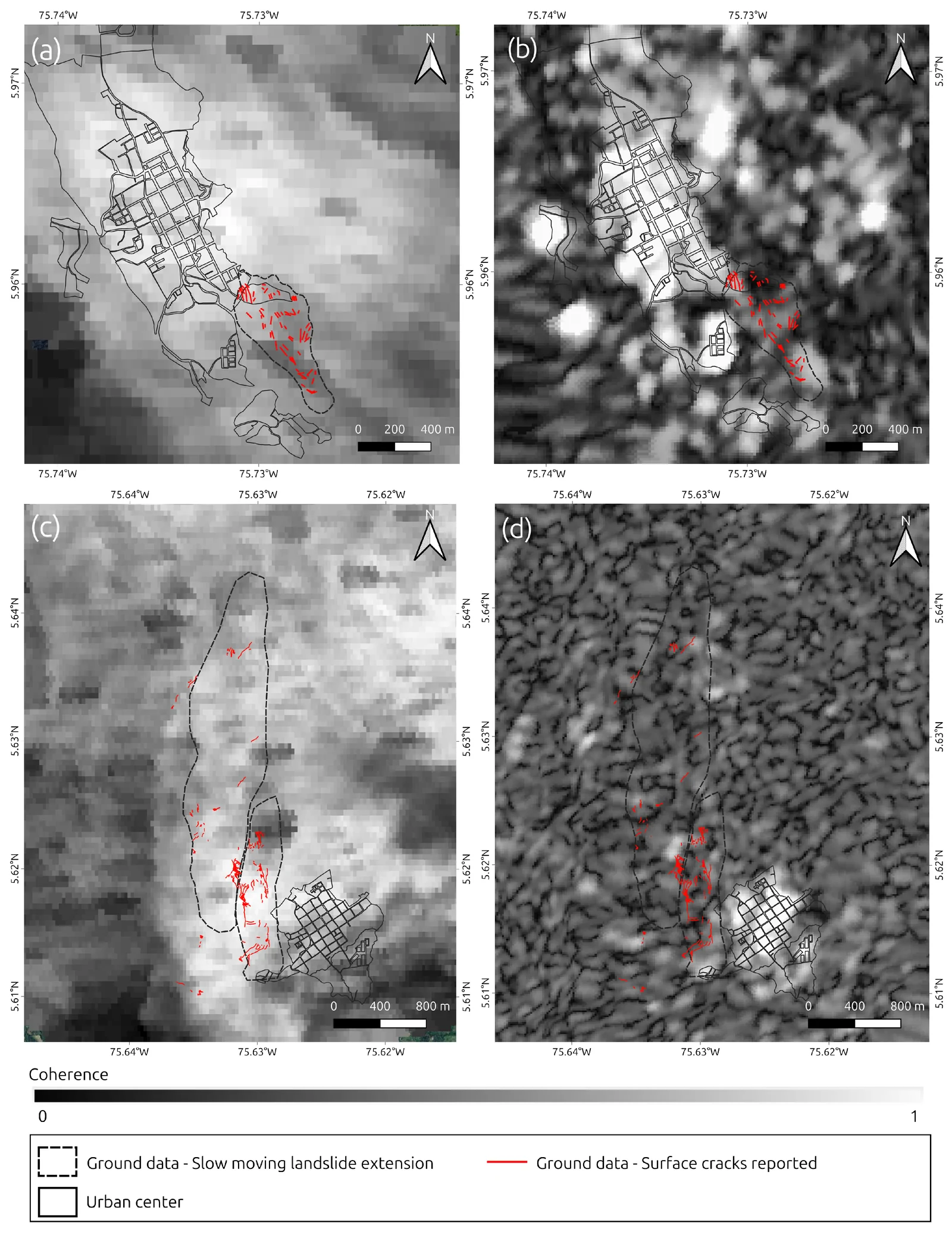
Coherence. (**a**) SAOCOM-1 in the Venecia study area. (**b**) Sentinel-1 in the Venecia study area. (**c**) SAOCOM-1 in the Valparaiso study area. (**d**) Sentinel-1 in the Valparaiso study area.

**Figure 13:**
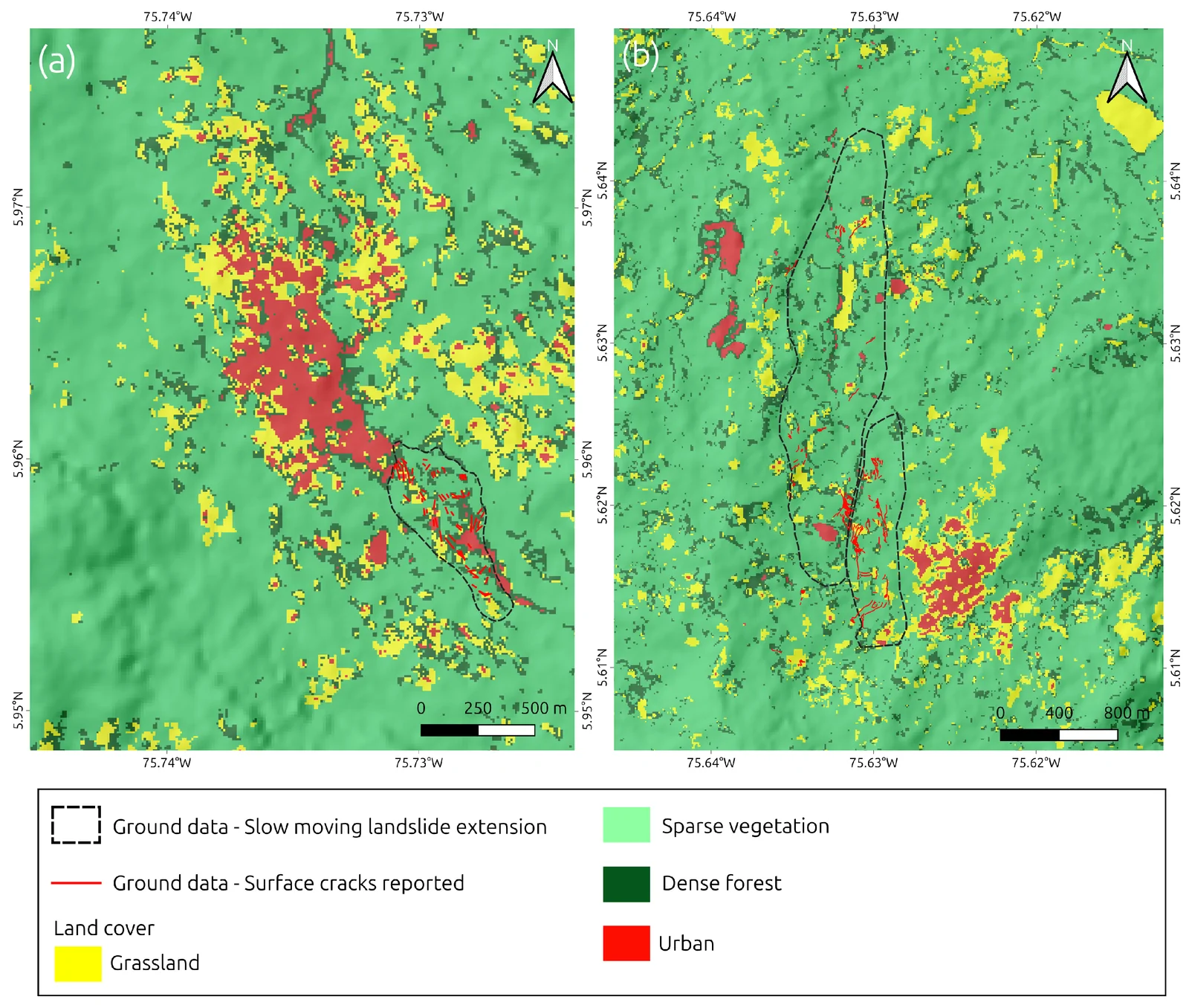
Land cover map. (**a**) Venecia study area. (**b**) Valparaiso study area.

**Figure 14:**
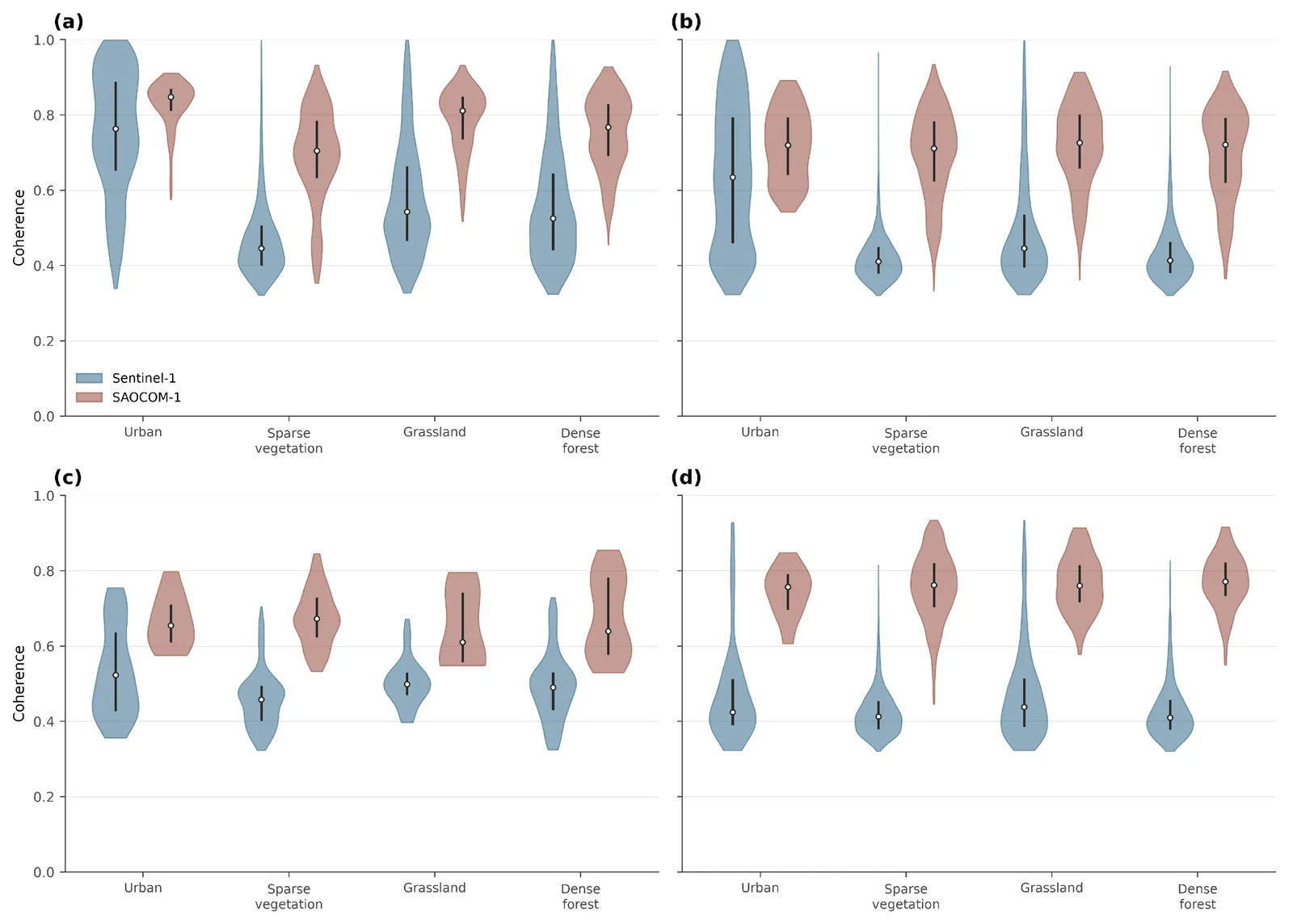
Coherence distribution by land cover class for Sentinel-1 (blue) and SAOCOM-1 (red). (**a**) Venecia and (**b**) Valparaíso over the total study area; (**c**) Venecia and (**d**) Valparaíso within the landslide body.

**Figure 15:**
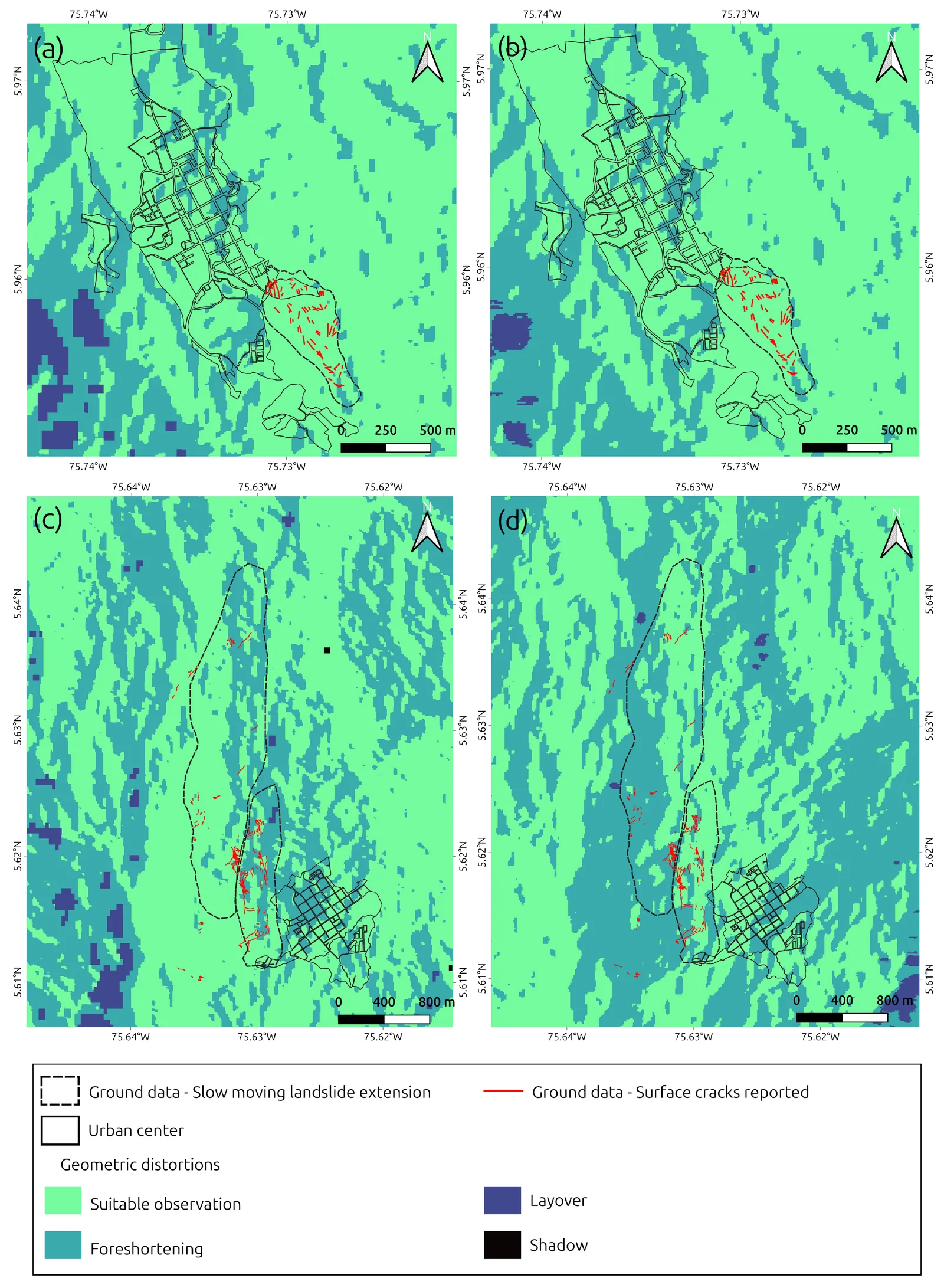
Geometric distortion maps for descending orbit: (**a**) SAOCOM-1 in the Venecia study area; (**b**) Sentinel-1 in the Venecia study area; (**c**) SAOCOM-1 in the Valparaíso study area; and (**d**) Sentinel-1 in the Valparaíso study area.

**Figure 16:**
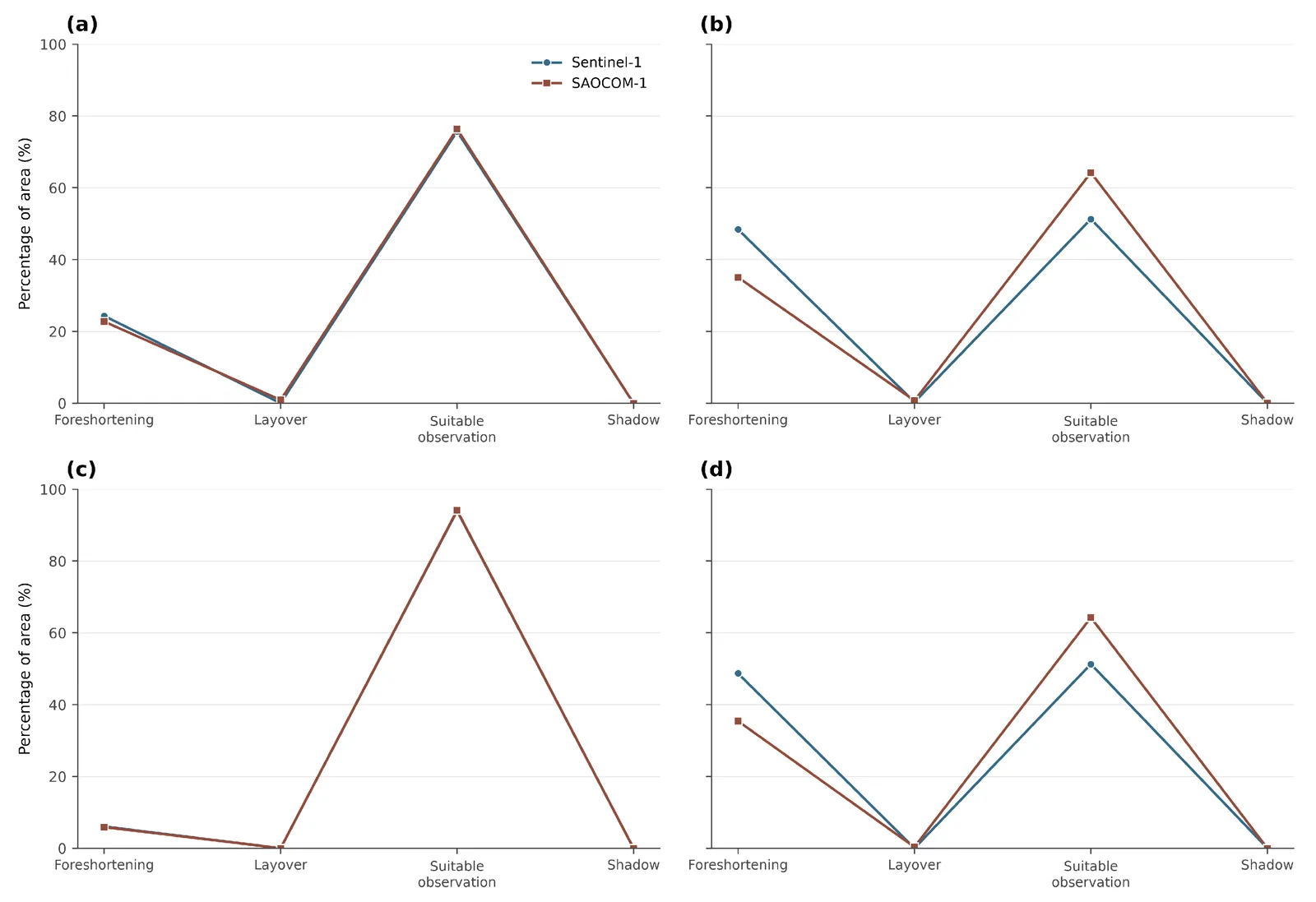
Percentage of area affected by each geometric distortion class for Sentinel-1 and SAOCOM-1. Panels (**a**,**b**) correspond to the total study area, whereas panels (**c**,**d**) correspond to the mapped landslide body in Venecia (**left**) and Valparaíso (**right**).

**Table 1:** Main parameters of SAR data.

Parameters	Sentinel-1	SAOCOM-1
Orbit track	Descending	Descending
Wavelength (cm)	5.6	23.6
Band	C	L
Constellation revisit time (d)	$6^{a}$	8
Range × azimuth resolution (m)	5×20	10×6
Polarization	Dual	Quad
Incidence angle (°)	31–46	27–30
Observation mode	IW	SM
Number of data	58	32
Date range	January 2024–August 2025	January 2024–August 2025

^*a*^ The 6-day revisit time corresponds to the nominal Sentinel-1A/Sentinel-1B constellation. During the study period, Sentinel-1B was not operational, and the effective acquisition interval of the dataset used in this study was 12 days.

**Table 2:** Coherence statistics over the total study area of each site. Pixel counts are not directly comparable between sensors, since the pixel spacing of the two products differs.

Study Area	Sensor	Mean	Median	Valid Pixels	Pixels γ≥ 0.4	Valid (%)
Valparaiso	Sentinel-1	0.43	0.41	39,818	24,142	60.63
Valparaiso	SAOCOM-1	0.69	0.71	7426	7384	99.43
Venecia	Sentinel-1	0.52	0.47	14,368	11,706	81.47
Venecia	SAOCOM-1	0.73	0.75	2266	2237	98.72

**Table 3:** Land cover composition of the total study area and of the landslide body at each site, derived from the unsupervised Sentinel-2 classification.

Land Cover	Venecia				Valparaiso			
	Total Study Area		Landslide Body		Total Study Area		Landslide Body	
	ha	%	ha	%	ha	%	ha	%
Urban	40.1	11.5	1.3	6.6	33.3	3.3	3.4	1.7
Sparse vegetation	202.3	58.1	10.7	54.0	773.9	77.3	153.9	77.3
Grassland	55.7	16.0	1.8	9.2	99.5	9.9	16.5	8.3
Dense forest	50.0	14.4	6.0	30.1	94.8	9.5	25.2	12.6

**Table 4:** Median coherence by land cover class for Sentinel-1 and SAOCOM-1 over the total study area and within the landslide body at each site.

Site	Land Cover	Total Study Area		Landslide Body	
		Sentinel-1	SAOCOM-1	Sentinel-1	SAOCOM-1
Venecia	Urban	0.76	0.85	0.52	0.66
	Sparse vegetation	0.45	0.71	0.46	0.67
	Grassland	0.54	0.81	0.50	0.61
	Dense forest	0.53	0.77	0.49	0.64
Valparaíso	Urban	0.63	0.72	0.43	0.76
	Sparse vegetation	0.41	0.71	0.41	0.77
	Grassland	0.44	0.73	0.44	0.76
	Dense forest	0.41	0.72	0.41	0.77

## Data Availability

Data availability on request.

## References

[1] Petley D. (2012). Global patterns of loss of life from landslides. Geology.

[2] Gariano S.L., Guzzetti F. (2016). Landslides in a changing climate. Earth-Sci. Rev..

[3] Fu L., Zhang Q., Wang T., Li W., Xu Q., Ge D. (2022). Detecting slow-moving landslides using InSAR phase-gradient stacking and deep-learning network. Front. Environ. Sci..

[4] Deng J., Dai K., Liang R., Chen L., Wen N., Zheng G., Xu H. (2023). Interferometric Synthetic Aperture Radar applicability analysis for potential landslide identification in steep mountainous areas with C/L band data. Remote Sens..

[5] Oven K.J. (2009). Landscape, Livelihoods and Risk: Community Vulnerability to Landslides in Nepal. Ph.D. Thesis.

[6] Marmoni G.M., Antonielli B., Caprari P., Di Renzo M.E., Marini R., Mastrantoni G., Mazzanti P., Patelli D., Bozzano F. (2025). Semi-automatic ranking of landslide candidate areas at a regional scale using EGMS InSAR data for territorial planning and risk management. Landslides.

[7] Hungr O., Leroueil S., Picarelli L. (2014). The Varnes classification of landslide types, an update. Landslides.

[8] Bekaert D.P.S., Handwerger A.L., Agram P., Kirschbaum D.B. (2020). InSAR-based detection method for mapping and monitoring slow-moving landslides in remote regions with steep and mountainous terrain: An application to Nepal. Remote Sens. Environ..

[9] Dai K.R., Shi X.L., Gou J.S. (2020). Diagnosing subsidence geohazard at Beijing Capital International Airport, from high-resolution SAR interferometry. Sustainability.

[10] Dong J., Liao M.S., Xu Q. (2018). Detection and displacement characterization of landslides using multi-temporal satellite SAR interferometry: A case study of Danba County in the Dadu River Basin. Eng. Geol..

[11] Dai C., Li W., Wang D., Lu H., Xu Q., Jian J. (2021). Active landslide detection based on Sentinel-1 data and InSAR technology in Zhouqu County, Gansu Province, Northwest China. J. Earth Sci..

[12] Guzzetti F., Mondini A.C., Cardinali M., Fiorucci F., Santangelo M., Chang K.-T. (2012). Landslide inventory maps: New tools for an old problem. Earth-Sci. Rev..

[13] Mondini A.C. (2021). Measures of spatial autocorrelation changes in multitemporal SAR images for event landslides detection. Remote Sens..

[14] Handwerger A.L., Fielding E.J., Sangha S.S., Bekaert D.P.S. (2022). Landslide sensitivity and response to precipitation changes in wet and dry climates. Geophys. Res. Lett..

[15] Rucci A., Ferretti A., Monti-Guarnieri A., Rocca F. (2019). Sentinel-1 SAR interferometry applications for landslide monitoring. Remote Sens..

[16] Wang T., Perissin D., Rocca F., Liao M. (2020). Time-series InSAR analysis for monitoring slow-moving landslides in mountainous terrain. ISPRS J. Photogramm. Remote Sens..

[17] Pepe A., Calò F. (2017). A review of interferometric synthetic aperture RADAR (InSAR) multi-track approaches for the retrieval of earth’s surface displacements. Appl. Sci..

[18] Berardino P., Fornaro G., Lanari R., Sansosti E. (2002). A new algorithm for surface deformation monitoring based on small baseline differential SAR interferograms. IEEE Trans. Geosci. Remote Sens..

[19] Wasowski J., Bovenga F., Dijkstra T., Meng X., Nutricato R., Chiaradia M.T. (2014). Persistent Scatterers Interferometry provides insight on slope deformations and landslide activity in the mountains of Zhouqu, Gansu, China. Landslide Science for a Safer Geoenvironment.

[20] Yalvac S. (2020). Validating InSAR-SBAS results by means of different GNSS analysis techniques in medium- and high-grade deformation areas. Environ. Monit. Assess..

[21] Lu H., Li W., Xu Q., Dong X., Dai C., Wang D. (2019). Early detection of landslides in the upstream and downstream areas of the Baige landslide, the Jinsha River based on optical remote sensing and InSAR technologies. Geomat. Inf. Sci. Wuhan Univ..

[22] Casagli N., Cigna F., Bianchini S., Hölbling D., Füreder P., Righini G., Del Conte S., Friedl B., Schneiderbauer S., Iasio C. (2016). Landslide mapping and monitoring by using radar and optical remote sensing: Examples from the EC-FP7 project SAFER. Remote Sens. Appl. Soc. Environ..

[23] Seppi S.A., López-Martinez C., Joseau M.J. (2022). Assessment of L-Band SAOCOM InSAR coherence and its comparison with C-Band: A case study over managed forests in Argentina. Remote Sens..

[24] Chowdhury T.A., Thiel C., Schmullius C. (2014). Growing stock volume estimation from L-band ALOS PALSAR polarimetric coherence in Siberian forest. Remote Sens. Environ..

[25] Sepúlveda S.A., Petley D.N. (2015). Regional trends and controlling factors of fatal landslides in Latin America and the Caribbean. Nat. Hazards Earth Syst. Sci..

[26] Aristizábal E., Yamamoto S. (2006). Geomorphology applied to landslide occurrence in the Aburrá Valley. Dyna.

[27] Aristizábal E., Martínez-Carvajal H., García-Aristizábal E. (2016). Modelling shallow landslides triggered by rainfall in tropical and mountainous basins. Eng. Geol..

[28] Poveda G., Álvarez D.M., Rueda Ó.A. (2011). Hydro-climatic variability over the Andes of Colombia associated with ENSO: A review of climatic processes and their impact on one of the Earth’s most important biodiversity hotspots. Clim. Dyn..

[29] Poveda G., Bedoya M., Aristizábal E. (2005). Mountain tropical rainfall: Evidence of phase-locking between the diurnal, annual and interannual cycles in the Andes of Colombia. Geophys. Res. Lett..

[30] Departamento Administrativo Nacional de Estadística (DANE) (2018). Censo Nacional de Población y Vivienda 2018.

[31] Mesa O., Poveda G., Vélez-Upegui J.I., Mejía-Valencia J.F., Hoyos-Ortiz C.D., Mantilla-Gutiérrez R., Barco-Mejía O.J., Cuartas-Pineda L.A., Botero-Hernández B., Montoya M. (2000). Distribución espacial y ciclos anual y semi-anual de la precipitación en Colombia. Proceedings of the XIV Seminario Nacional de Hidráulica e Hidrología.

[32] Poveda G. (2004). La hidroclimatología de Colombia: Una síntesis desde la escala interdecadal hasta la escala diurna. Rev. Acad. Colomb. Cienc. Exactas Fis. Nat..

[33] Aristizábal E., Sánchez O. (2020). Spatial and temporal patterns and the socioeconomic impacts of landslides in the tropical and mountainous Colombian Andes. Disasters.

[34] Cediel J.E., Shaw R.P., Cáceres C., Bartolini C., Buffler R.T., Blickwede J. (2003). Tectonic assembly of the Northern Andean Block. The Circum-Gulf of Mexico and the Caribbean: Hydrocarbon Habitats, Basin Formation, and Plate Tectonics.

[35] Ramos V.A. (1999). Plate tectonic setting of the Andean Cordillera. Episodes.

[36] Restrepo J.J., Toussaint J.F. (1988). Terranes and continental accretion in the Colombian Andes. Episodes.

[37] González H., Núñez A., Paris G. (1988). Mapa Geológico de Colombia: Memoria Explicativa.

[38] Aspden J.A., McCourt W.J., Brook M. (1987). Geometrical control of subduction-related magmatism: The Mesozoic and Cenozoic plutonic history of Western Colombia. J. Geol. Soc..

[39] Grosse E. (1926). El Terciario Carbonífero de Antioquia.

[40] Silva-Tamayo J.C., Lara M., Salazar-Franco A.M., Gómez J., Pinilla-Pachon A.O. (2020). Oligocene–Miocene coal-bearing successions of the Amagá Formation, Antioquia, Colombia: Sedimentary environments, stratigraphy and tectonic implications. The Geology of Colombia, Volume 3—Paleogene–Neogene.

[41] Lara M., Salazar-Franco A.M., Silva-Tamayo J.C. (2018). Provenance of the Cenozoic siliciclastic intramontane Amagá Formation: Implications for the early Miocene collision between Central and South America. Sediment. Geol..

[42] Ordóñez-Carmona O., Pimentel M.M., Restrepo J.J. (2007). Geochronological and isotopic constraints on the Miocene Combia volcanic arc, Central Cordillera of Colombia. J. S. Am. Earth Sci..

[43] Servicio Geológico Colombiano (2014). Memoria Explicativa de la Zonificación de la Susceptibilidad y la Amenaza Relativa por Movimientos en Masa, Escala 1:100.000, Plancha 186—Riosucio.

[44] Universidad EAFIT (2025). Evaluación de la Susceptibilidad y Amenaza por Movimiento en Masa y Avenida Torrencial en el Cerro Bravo y Estudio de Detalle de la Amenaza por Movimiento en Masa en los Sectores Santa Ana y La Corona, y por Inundación y Avenida Torrencial de las Corrientes La Tigra, La Galápago y Santa Bárbara, Casco Urbano de Venecia, Antioquia.

[45] Universidad Nacional de Colombia, Sede Medellín (2023). Estudios de Amenaza a Nivel de Detalle en el Sector Sabaletas del Municipio de Valparaíso, Antioquia, para Establecer las Áreas en Condición de Amenaza y Riesgo.

[46] Farr T.G., Rosen P.A., Caro E., Crippen R., Duren R., Hensley S., Kobrick M., Paller M., Rodriguez E., Roth L. (2007). The Shuttle Radar Topography Mission. Rev. Geophys..

[47] Lanari R., Mora O., Manunta M., Mallorqui J.J., Berardino P., Sansosti E. (2004). A small-baseline approach for investigating deformations on full-resolution differential SAR interferograms. IEEE Trans. Geosci. Remote Sens..

[48] Mora O., Mallorquí J.J., Broquetas A. (2003). Linear and non-linear terrain deformation maps from a reduced set of interferometric SAR images. IEEE Trans. Geosci. Remote Sens..

[49] Zhang J., Zuo X., Li Y. (2025). Detection and assessment of potential landslides in the Xiaojiang River Basin using SBAS-InSAR. Sci. Rep..

[50] Carballo F.D., Kaufman J.F., Wilson C.G.J., Pardo-Duró M.L., Wright E.M. (2025). Application of DInSAR technique for landslide detection at the entrance of Aconcagua Provincial Park, Mendoza, Argentina. J. S. Am. Earth Sci..

[51] Zhou C., Cao Y., Hu X., Yin K., Wang Y., Catani F. (2022). Enhanced dynamic landslide hazard mapping using MT-InSAR method in the Three Gorges Reservoir Area. Landslides.

[52] Jia H., Wang Y., Ge D., Deng Y., Wang R. (2022). InSAR Study of Landslides: Early Detection, Three-Dimensional, and Long-Term Surface Displacement Estimation—A Case of Xiaojiang River Basin, China. Remote Sens..

[53] Rosen P.A., Gurrola E., Sacco G.F., Zebker H. The InSAR scientific computing environment. Proceedings of the EUSAR 2012—9th European Conference on Synthetic Aperture Radar.

[54] Pechnikov A. (2024). PyGMTSAR (Python InSAR), version 2024.2.8.

[55] Yunjun Z., Fattahi H., Amelung F. (2019). Small baseline InSAR time series analysis: Unwrapping error correction and noise reduction. Comput. Geosci..

[56] Lundgren P., Usai S., Sansosti E., Lanari R., Tesauro M., Fornaro G., Berardino P. (2001). Modeling surface deformation observed with SAR interferometry at Campi Flegrei Caldera. J. Geophys. Res..

[57] Usai S. (2001). A New Approach for Long Term Monitoring of Deformations by Differential SAR Interferometry. Ph.D. Thesis.

[58] Yu C., Li Z., Penna N.T., Crippa P. (2018). Generic atmospheric correction model for interferometric synthetic aperture radar observations. J. Geophys. Res. Solid Earth.

[59] Wu R., Liu G., Bao X., Lv J., Shama A., Zhang B., Mao W., Chen J., Yang Z., Zhang R. (2025). Eliminating geometric distortion with dual-orbit Sentinel-1 SAR fusion for accurate glacial lake extraction in Southeast Tibet Plateau. Int. J. Appl. Earth Obs. Geoinf..

[60] Simons M., Rosen P. (2007). Interferometric synthetic aperture radar geodesy. Geodesy.

[61] Huang C., Wang L., Zhao L., Liu S., Zou D., Liu G., Hu G., Du E., Xiao Y., Wang C. (2024). Potential of C-band Sentinel-1 InSAR for ground surface deformation monitoring in the southern boreal forest: An investigation in the Genhe River basin. Int. J. Appl. Earth Obs. Geoinf..

[62] Zanaga D., Van De Kerchove R., Daems D., De Keersmaecker W., Brockmann C., Kirches G., Wevers J., Cartus O., Santoro M., Fritz S. (2022). ESA WorldCover 10 m 2021 v200.

